# Collagen analogs with phosphorylcholine are inflammation-suppressing scaffolds for corneal regeneration from alkali burns in mini-pigs

**DOI:** 10.1038/s42003-021-02108-y

**Published:** 2021-05-21

**Authors:** Fiona C. Simpson, Christopher D. McTiernan, Mohammad Mirazul Islam, Oleksiy Buznyk, Philip N. Lewis, Keith M. Meek, Michel Haagdorens, Cindy Audiger, Sylvie Lesage, François-Xavier Gueriot, Isabelle Brunette, Marie-Claude Robert, David Olsen, Laura Koivusalo, Aneta Liszka, Per Fagerholm, Miguel Gonzalez-Andrades, May Griffith

**Affiliations:** 1 Centre de recherche, Hôpital Maisonneuve-Rosemont, Montréal, QC Canada; 2 Départment d’Ophtalmologie, Université de Montréal, Montréal, QC Canada; 3 Institut du Génie Biomédicale, Université de Montréal, Montréal, QC Canada; 4 Centre de recherche du Centre hospitalier de l’Université de Montréal, Montréal, QC Canada; 5 Division of Cardiac Surgery, University of Ottawa Heart Institute, Ottawa, ON Canada; 6 Disruptive Technology Laboratory, Massachusetts Eye and Ear and Schepens Eye Research Institute, Department of Ophthalmology, Harvard Medical School, Boston, MA USA; 7 Institute for Clinical and Experimental Medicine, Linköping University, Linköping, Sweden; 8 Filatov Institute of Eye Diseases and Tissue Therapy of the NAMS of Ukraine, Odessa, Ukraine; 9 School of Optometry and Vision Sciences, Cardiff University, Cardiff, UK; 10 Department of Ophthalmology, Visual Optics and Visual Rehabilitation, University of Antwerp, Antwerp, Belgium; 11 Département de microbiologie, infectiologie et immunologie, Université de Montréal, Montréal, QC Canada; 12 Department of Ophthalmology, Valence Hospital, Valence, France; 13 FibroGen Inc., San Francisco, CA USA; 14 Faculty of Medicine and Health Technology, Tampere University, Tampere, Finland; 15 Maimonides Biomedical Research Institute of Cordoba (IMIBIC), Department of Ophthalmology, Reina Sofia University Hospital and University of Cordoba, Cordoba, Spain

**Keywords:** Translational research, Implants

## Abstract

The long-term survival of biomaterial implants is often hampered by surgery-induced inflammation that can lead to graft failure. Considering that most corneas receiving grafts are either pathological or inflamed before implantation, the risk of rejection is heightened. Here, we show that bioengineered, fully synthetic, and robust corneal implants can be manufactured from a collagen analog (collagen-like peptide-polyethylene glycol hybrid, CLP-PEG) and inflammation-suppressing polymeric 2-methacryloyloxyethyl phosphorylcholine (MPC) when stabilized with the triazine-based crosslinker 4-(4,6-Dimethoxy-1,3,5-triazin-2-yl)-4-methylmorpholinium chloride. The resulting CLP-PEG-MPC implants led to reduced corneal swelling, haze, and neovascularization in comparison to CLP-PEG only implants when grafted into a mini-pig cornea alkali burn model of inflammation over 12 months. Implants incorporating MPC allowed for faster nerve regeneration and recovery of corneal sensation. CLP-PEG-MPC implants appear to be at a more advanced stage of regeneration than the CLP-PEG only implants, as evidenced by the presence of higher amounts of cornea-specific type V collagen, and a corresponding decrease in the presence of extracellular vesicles and exosomes in the corneal stroma, in keeping with the amounts present in healthy, unoperated corneas.

## Abstract (web summary)

Simpson, McTiernan et al. develop fully synthetic corneal implants. The resulting CLP-PEG-MPC implants show reduced corneal swelling, haze, and neovascularization compared to CLP-PEG only implants when grafted into a mini-pig cornea alkali burn model of inflammation over 12 months. This study suggests the superiority of CLP-PEG-MPC implants over the CLP-PEG only implants.

## Introduction

Biomaterial implants, like organ transplants, suffer inflammation that can result in immune rejection and graft failure.^1^ This is a consideration when developing corneal implants for treating patients not amenable to conventional human donor transplantation.

Globally, approximately 23 million people have unilateral corneal blindness and 4.6 million are bilaterally blind.^2^ For the past century, the only widely accepted treatment for corneal blindness has been human donor corneal transplantation. First-time, low-risk grafts are over 90% successful for the first 2 years post-operation.^3^ However, this declines to 55% by 15 years due to chronic inflammation.^4^ A severe worldwide human donor cornea shortage leaves 12.7 million patients awaiting transplantation, with only one in 70 patients being treated.^5^ Patients with inflammation and severe pathologies have risks of up to 70% for rejecting donor allografts.^3^ So, they often remain untreated, with valuable tissues allocated to patients with better chances of success.^6^

Artificial corneas or keratoprostheses (KPros) were developed to treat high-risk patients, but most have failed due to adverse biomaterial-induced host reactions. The most successful design currently in clinical application, the Boston KPro, has a poly(methyl methacrylate)(PMMA) optic cylinder that allows light transmission into the eye for vision. However, human corneal tissue is needed as a carrier for implantation. The graft-host tissue interface has been implicated in the formation of retroprosthetic membrane,^7^ corneal tissue melt (keratolysis) and tractional retinal detachment. Further, the issues of PMMA-induced inflammation remain^8^ and potentially contribute to periprosthetic keratolysis and KPro extrusion.^9^ In a mouse model, it was shown that inflammatory cytokines, TNF-α and IL-1, elicited by Boston KPro implantation can result in optic nerve damage.^10^ Hence, KPros are only used for end-stage eyes.

Diverse anti-inflammatory approaches have been developed to improve biocompatibility and integration of biomaterial implants. Many of these involve surface modification of the biomaterials.^11^ Introduction of topographical features on surfaces were reported to reduce adherence of macrophages,^12^ and alter the profile of cytokines produced in vivo in rats. Other strategies involve converting hydrophobic surfaces that promote inflammatory reactions (e.g., increased leukocyte adhesion, macrophage fusion, and pro-inflammatory cytokine release^13,14^) to more tolerogenic hydrophilic ones by modifying surface chemistries.^11,13^ Hydrophilic terminal groups (NH_2_, OH, COOH) were shown to temper macrophage conversion into foreign body giant cells that characterize adverse reactions, and decrease expression of pro-inflammatory cytokines.^11^ Inflammation-decreasing surface coatings include anti-fouling molecules such as polyethylene glycol (PEG) that prevent non-specific cell adhesion, and anti-inflammatory molecules like glycosaminoglycans, steroids (e.g., dexamethasone), α-melanocyte-stimulating hormone, and interleukin-1 receptor antagonists.^11^

Corneal transplantation, particularly in high-risk cases, can trigger allogenic sensitization against foreign cells, releasing inflammatory chemokines/cytokines that mediate the recruitment and activation of immune cells including antigen-presenting dendritic cells to the graft.^15,16^ Host dendritic cells are exposed to shed donor antigen as they migrate into the graft. As the dendritic cells process the alloantigens, they drain via the lymphatic system to local lymph nodes where they activate naive T-cells that are involved in rejection.^15^ The inflammatory cycle triggered by dendritic cell activation also results in lymphangiogenesis and angiogenesis, which in turn enhances the sensitization to alloantigens.^17^ Implants that do not activate dendritic cells at the outset would therefore be optimal.

To address corneal donor shortage and circumvent immune problems, Fagerholm et al. developed corneal implants made from carbodiimide-crosslinked recombinant human collagen type III (RHCIII) and grafted 500 µm thick implants into 10 patients.^18,19^ Being cell-free, the implants were immune compatible, did not activate dendritic cells, and supported the stable regeneration of corneal epithelium, stroma, and nerves.^19,20^ Polymeric 2-methacryloyloxyethyl phosphorylcholine (MPC) was incorporated into implants to modulate the inflammation in corneas of patients at high-risk of graft rejection and therefore not prioritized for donor corneal transplantation.^21^ Partial-thickness RHCIII-MPC grafted into high-risk corneas after removing active ulcers or scars promoted stable corneal epithelium and stromal regeneration over the averaged 24-month observational period.^21^ It was subsequently shown that MPC-containing RHCIII hydrogels do not activate dendritic cells, but instead induce dendritic cell apoptosis.^20^

While RHCIII-based implants performed well in clinical trials, RHCIII is a large macromolecule with manufacturing challenges. Replicating full-length native collagen, RHCIII contains numerous 4-hydroxyproline amino acids for stable triple helix formation. Therefore, to produce RHCIII, it is not only necessary to produce the collagen but also prolyl 4-hydroxylase, the enzyme that catalyzes 4-hydroxyproline formation from proline.^22^ In addition, recombinant pepsin is needed to cleave the telopeptides from the full-length protein prior to use.^23^ This means the expression of three different complex proteins is required to produce RHCIII. Short, self-assembling collagen-like peptides (CLPs) have been developed by several groups as alternatives to native collagen.^24^ As short peptides, they can be produced synthetically, are easy to purify and also easy to manipulate and customize for different applications.^24,25^ A 36 amino-acid CLP was developed by the Hartgerink group as a collagen analog,^26^ and performed well as a hemostat.^27^ When conjugated to a multi-arm polyethylene glycol (PEG) through a short peptide and thiol-maleimide, the resulting CLP-PEG hydrogel could be fabricated into corneal implants that promoted regeneration in the corneas of mini-pigs.^28,29^ However, the N-(3- dimethylaminopropyl)-N′-ethylcarbodiimide hydrochloride (EDC) used for stabilizing the hydrogels was possibly pro-inflammatory.^20,30^

As mentioned above there are currently no or limited treatment options for patients awaiting corneal transplantation that are at high-risk of graft rejection. Our goal was thus to bioengineer fully synthetic, robust and easy to manufacture corneal implants, as alternatives to human donor corneas or prostheses. In addition to a simple manufacturing process, the implants must promote tolerogenic properties or limit inflammation while stimulating stable regeneration of corneal tissues and nerves, to be amenable for use in high-risk patients. Hence, we improved on CLP-PEG implants by stabilizing with 4-(4,6-Dimethoxy-1,3,5-triazin-2-yl)-4-methylmorpholinium chloride (DMTMM) a triazine-based crosslinker, as opposed to using the pro-inflammatory EDC stabilizer. Indeed, DMTMM has a much lower toxicity compared to EDC and its commonly used co-reactant, N-hydroxy-succinimide (NHS).^31^ We further modified the CLP-PEG implants by including inflammation-suppressing MPC. The CLP-PEG-MPC implants were characterized and compared to those without MPC in vitro and after grafting into the corneas of mini-pigs. Inflammation and severe pathological conditions were simulated by a standard alkali burn cornea model.

## Results

### Hydrogel manufacture and characterization

**Fig. 1:**
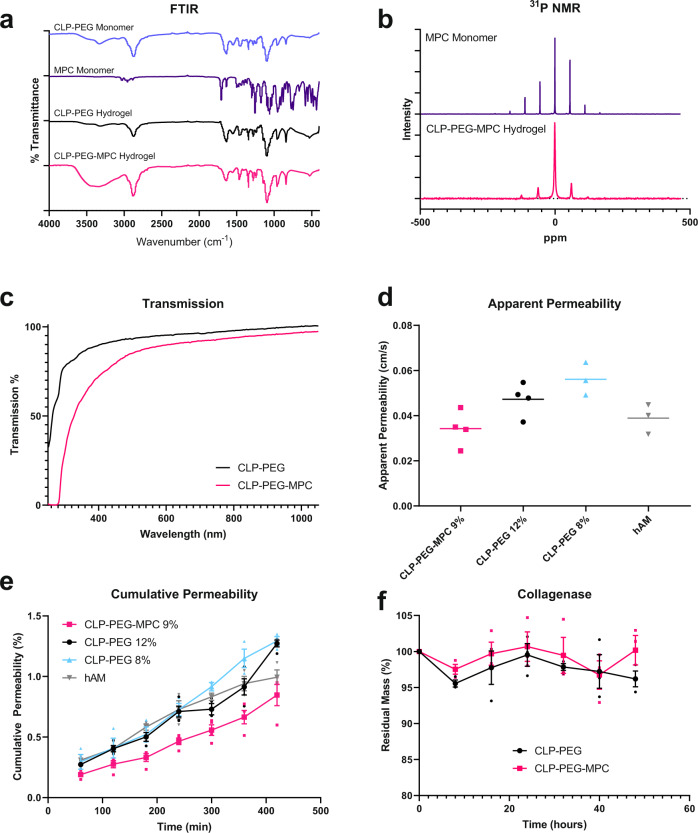
Chemical and optical analyses of CLP-PEG-MPC and CLP-PEG hydrogels. **a** Fourier transformed infrared spectroscopy shows the incorporation of the CLP-PEG monomer (lavender line) into the CLP-PEG hydrogel (black line) the CLP-PEG monomer and MPC monomer (purple line) into the CLP-PEG-MPC hydrogel (pink line). **b** ^31^P NMR spectroscopy with peaks that show the incorporation of phosphorylcholine (purple line) into CLP-PEG-MPC hydrogels (pink line) (*n*=1, per group). **c** Both CLP-PEG (black line) and CLP-PEG-MPC (pink line) hydrogels transmit the full spectrum of visible light, but CLP-PEG-MPC blocks short UV wavelengths (*n*=1, per group). **d**, **e** Apparent and cumulative permeability of 700 Da Alexa Fluor^®^ 568 hydrazide sodium salt through CLP-PEG-MPC hydrogels (pink squares, *n*=4 technical replicates (TR)) in comparison to 12 and 8% CLP-PEG (black circles, *n*=4; light blue triangles, *n*=3 TR, respectively) hydrogels and hAM (gray inverted triangles, *n*=3 TR). **f** Both CLP-PEG-MPC (pink squares) and CLP-PEG hydrogels (black circles) remained stable and minimally degraded when exposed to collagenase enzyme (*n*=3 TR per group).

Infrared spectroscopy (Fig. 1a) and ^31^P NMR spectroscopy (Fig. 1b) confirmed the incorporation of MPC into DMTMM-crosslinked CLP-PEG hydrogels that were fabricated into cornea-shaped implants, 10 mm in diameter and 500 µm thick. In particular, the CLP-PEG-MPC hydrogels showed a peak at approximately 0 ppm on ^31^P NMR, which was indicative of the ring opening generated by the addition of trimethylamine to the 2‐hydroxyethyl methacrylate (HEMA) and ethylene chlorophosphate intermediary during MPC synthesis (Fig. 1b).^32^ Both hydrogels were highly transparent in visible light (%T400 nm–700 nm > 80%) (Fig. 1c). However, CLP-PEG-MPC (Fig. 1c), but not CLP-PEG, hydrogels blocked up to 60% transmission of UV-A (300–400 nm wavelength).

Table 1 summarizes the implant properties and how they compare with RHCIII implants that were tested clinically, and to human corneas. Neither hydrogels nor the RHCIII comparator were as tough as the human cornea, although they were optically slightly superior. As the hydrogels contained over 90% water, their refractive indices approximated that of water (1.33). CLP-PEG hydrogels were stiffer (Young’s modulus of 0.150 ± 0.015 MPa) and less elastic (elongation at break 49.96 ± 8.10 %) than those incorporating MPC. CLP-PEG-MPC implants displayed a lower Young’s modulus (0.044 ± 0.010 MPa) and higher elongation at break (59.50 ± 7.70 %). Furthermore, rheology showed that the CLP-PEG hydrogels had a higher storage modulus (*G*′) (22.36 ± 1.489 kPa) than the CLP-PEG-MPC (15.15 ± 1.086 kPa) indicating an increased amount of structure present in the CLP-PEG only implants. However, considering the loss modulus of both gels was lower than the storage modulus, both hydrogels were considered ductile. Apparent and cumulative permeability measurements of the implants (Fig. 1d, e) showed no significant difference in the apparent or cumulative permeability amongst CLP-PEG-MPC, CLP-PEG, or the human amniotic membrane (hAM) control, which is the current gold standard for ocular surface reconstruction. Both CLP-PEG-MPC and CLP-PEG implants were highly resistant to bacterial collagenase degradation in vitro (Fig. 1f).

**Table 1:** Characterization of CLP-PEG-MPC hydrogels compared to CLP-PEG only hydrogels, biosynthetic implants currently in patients and the human cornea.

	CLP-PEG-MPC	CLP-PEG	Biosynthetic implants^a^	Human cornea
Tensile strength (MPa)	0.022 ± 0.004	0.56 ± 0.21	0.286 ± 0.06^19^	3.81 ± 0.40^48^
Elongation (%)	59.50 ± 7.70	49.96 ± 8.10	20.149 ± 7.614^19^	N/A
Young’s modulus (MPa)	0.044 ± 0.010	0.150 ± 0.015	1.749 ± 0.782^19^	3-13^49, 50^
Storage modulus (*G*′) (kPa)	15.15 ± 1.086	22.36 ± 1.489	–	–
Loss modulus (*G*″) (kPa)	0.1522 ± 0.0569	0.0433 ± 0.006	–	–
Transmission (%)	29-80(UV) 80-97(Vis)	32-92(UV) 92-99(Vis)	95.1 ± 0.05^19^	87.1 ± 2.0 (at 500 nm)^51^
Refractive index	1.340 ± 0.005	1.338 ± 0.004	1.3507 ± 0.0011^52^	1.423–1.436^53^
Water content (%)	90.94 ± 0.78	92.67 ± 0.85	91.5 ± 0.9^19^	78^54^
Residual mass (%) from collagenase Degradation at 48 h	100.17 ± 3.54	96.19 ± 1.89	<10%^52^	–

^**a**^RHCIII implants that have been stably grafted into 10 patients and showed regeneration at 4 years post-operation as reported in Fagerholm et al.^19^ The *n*=3 technical replicates per group for CLP-PEG-MPC, CLP-PEG and biosynthetic implants. Human cornea *n*-value varies by study.

### In vitro biocompatibility and immune compatibility

**Fig. 2:**
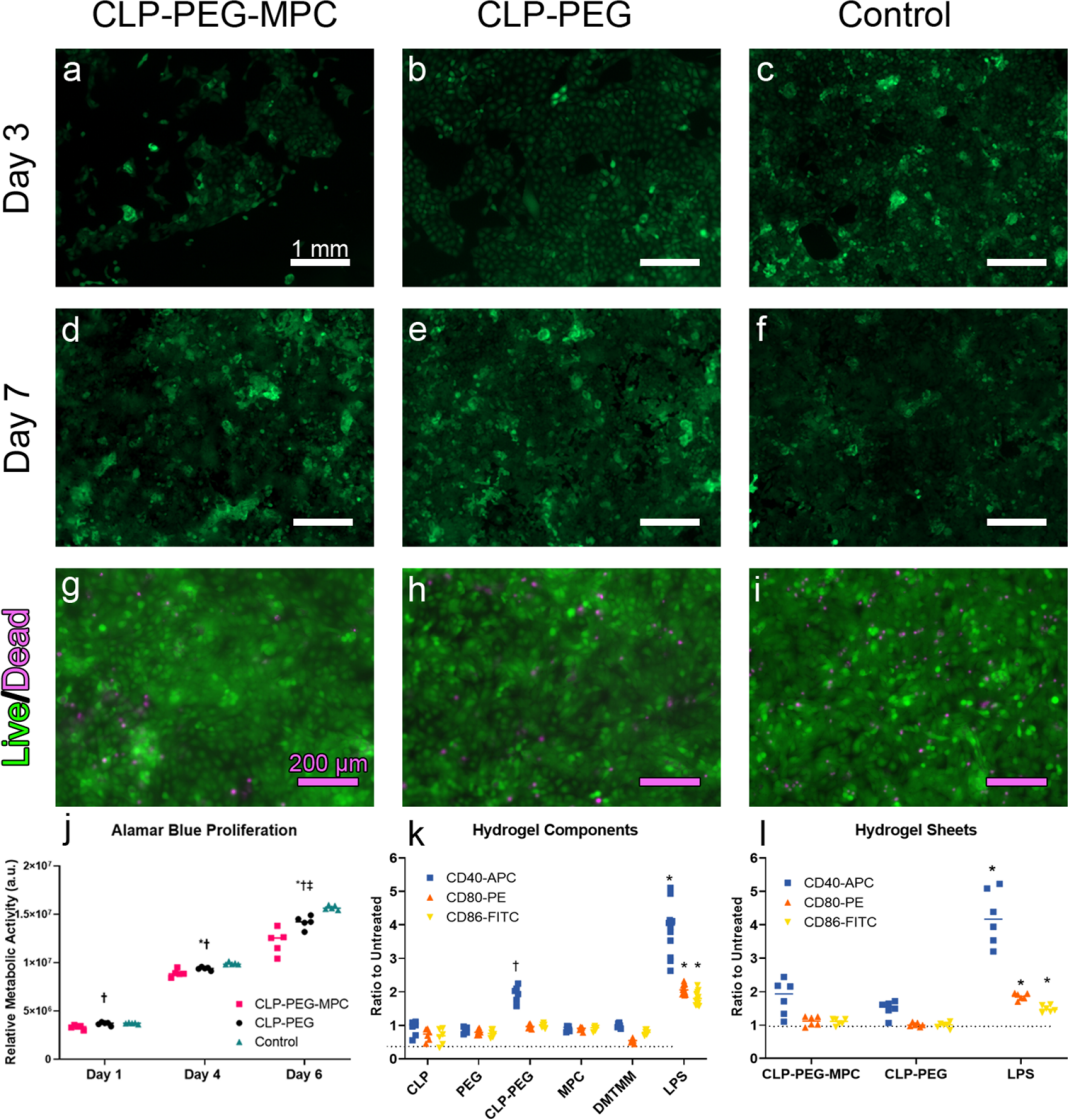
In vitro analyses of CLP-PEG-MPC and CLP-PEG hydrogels. **a**–**f** GFP-tagged human corneal epithelial cells proliferated on both CLP-PEG-MPC and CLP-PEG hydrogels over 7 days (*n*=4 per group). White scale bars, 1 mm. **g**–**i** Live/dead pictures of human corneal epithelial cells proliferated on both CLP-PEG-MPC and CLP-PEG hydrogels over 6 days (green represents live cells and magenta represents dead cells) (*n*=5 per group). Red scale bars, 200 µm. **j** Alamar Blue proliferation study of human corneal epithelial cells proliferated on CLP-PEG-MPC hydrogels (pink squares), CLP-PEG hydrogels (black circles) and tissue culture plate controls (aqua triangles) at different time points (*n*=5 TR per group). Analysis by one-way ANOVA with Tukey’s multiple comparisons test within each time point. **k** Culture of BMDCs on monomeric hydrogel components and crosslinkers showed no activation as determined by the significantly lower expression of co-stimulatory receptors CD40-APC (blue squares), CD80-PE (orange triangles) and CD86-FITC (yellow inverted triangles) compared to lipopolysaccharide (LPS) stimulation, a positive control for dendritic cell activation. Analysis by one-way ANOVA with Dunnett’s multiple comparisons test (*n*=6 biological replicates (BR) per group). *LPS vs component, *p*<0.0001, †CLP-PEG vs component, *p*<0.05. **l** CLP-PEG-MPC and CLP-PEG hydrogels do not upregulate CD40-APC (blue squares), CD80-PE (orange triangles) or CD86-FITC (yellow inverted triangles). One-way ANOVA with Tukey’s multiple comparisons test. *LPS vs hydrogel, *p*<0.0001.

Initial growth (1–6 days) of immortalized human corneal epithelial cells (HCECs) expressing green fluorescent protein (GFP) on CLP-PEG-MPC was slower than on CLP-PEG and tissue culture plastic controls (Fig. 2a–c), but at 7 days in culture, cells on both hydrogels were confluent (Fig. 2d, e). Live-dead staining showed very few dead cells, confirming that the hydrogels were non-cytotoxic (Fig. 2g, h). These observations were confirmed by an Alamar Blue proliferation study carried out with non-GFP-tagged HCECs (Fig. 2j; Supplementary Table 1). Bone-marrow-derived dendritic cell (BMDC) activation assays were conducted for the individual hydrogel components (Fig. 2k) and the completed hydrogels (Fig. 2l). The crosslinker EDC-NHS was compared to equimolar concentrations of DMTMM. However, EDC-NHS was cytotoxic and resulted in such low absolute cell counts of CD11c^+^ cells, that it was excluded from subsequent analysis. Of the structural hydrogel components, only conjugated CLP-PEG activated CD40 (Fig. 2k). Both hydrogels upregulated CD40 to levels above that of the untreated controls, but significantly lower than the lipopolysaccharide positive control level (*F*= 40.03, *p*<0.0001) (Fig. 2l).

### In vivo clinical evaluation of implants

**Fig. 3:**
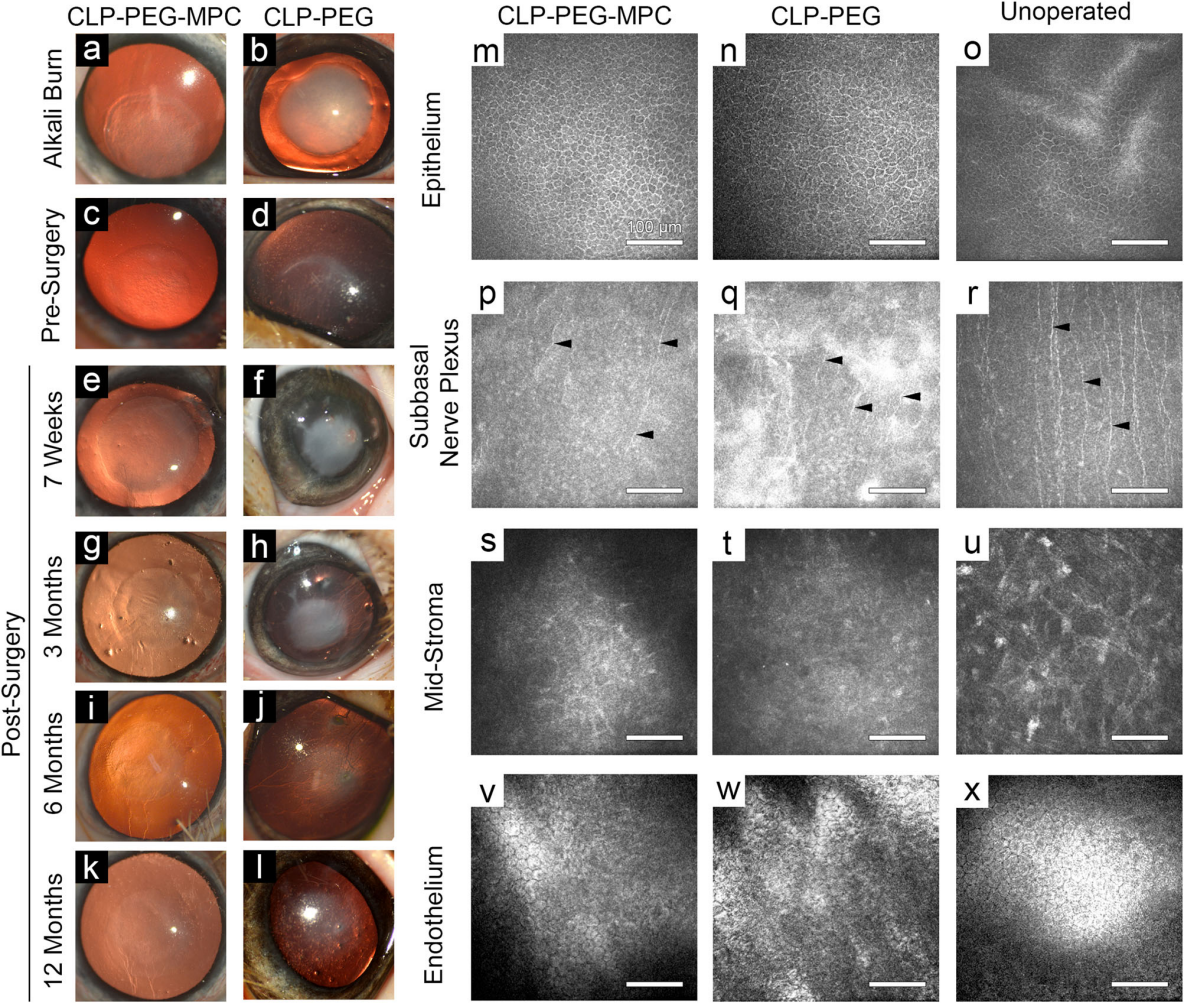
CLP-PEG-MPC implants in alkali burned mini-pig corneas compared to CLP-PEG only implants. **a**–**l** Surgical progression of CLP-PEG-MPC hydrogels compared to CLP-PEG hydrogels implanted in mini-pig corneas following alkali burns. Corneal haze was seen immediately after the alkali burn to the central cornea (**a**, **b**) and is still present just before surgery (**c**, **d**). After surgery, haze is present up to 6 months (**e**–**j**) and is decreased by 12 months post-operation (**k**–**l**). **m**–**u** In vivo confocal microscopy of the regenerated CLP-PEG-MPC and CLP-PEG neo-corneas at 12 months post-operation in comparison to unoperated corneas, showing regeneration of corneal epithelium, subepithelial nerves (arrowheads) and stroma. The unoperated endothelium (**v**–**x**) remains intact. White scale bars, 100 µm.

**Fig. 4:**
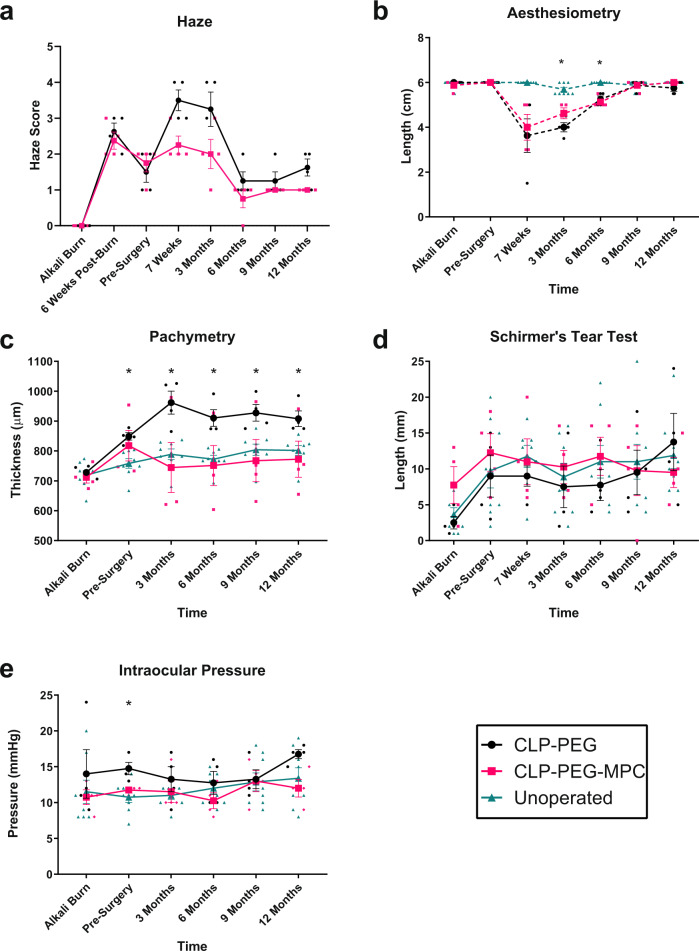
Clinical follow-up of regenerating neo-corneas after CLP-PEG and CLP-PEG-MPC implantation into post-alkali burned mini-pig corneas. **a** Corneal haze increased in response to alkali burn and surgery in CLP-PEG (black circles) and CLP-PEG-MPC (pink squares) but diminished over the 12-month follow-up period. **b** Corneal blink response measured by Cochet-Bonnet aesthesiometry showing immediate decrease post-surgery in CLP-PEG-MPC (pink squares) and CLP-PEG (black circles) with recovery to pre-operative levels by 12 months. Unoperated eyes (aqua triangles) do not show changes in the blink response. **c** CLP-PEG-MPC (pink squares) grafts resulted in a corneal thickness comparable to the unoperated eye (aqua triangles), but CLP-PEG (black circles) resulted in significant increases in corneal thickness. **d** Schirmer’s tear tests showing a decrease in tear production immediately post-burn due to trauma, followed by a normal tear production in CLP-PEG-MPC (pink squares) and CLP-PEG (black circles) compared to unoperated controls (aqua triangles). **e** Intraocular pressure was maintained within normal parameters CLP-PEG-MPC (pink squares), CLP-PEG (black circles) and unoperated (aqua triangles) corneas throughout all stages of follow-up. Data displayed as mean ± SEM. Statistical analyses by two-way repeat measures ANOVA with Geisser’s Greenhouse correction and a Tukey’s or Sidak multiple comparison test. *Unoperated vs CLP-PEG, *p*<0.05. †Unoperated vs. CLP-PEG-MPC, *p*<0.05. ‡ CLP-PEG vs. CLP-PEG-MPC, *p*<0.05. For all charts the unit of analysis is the eye: CLP-PEG *n*=4, CLP-PEG-MPC *n*=4, unoperated *n*=8.

In compliance with the Swedish Animal Welfare Ordinance and the Animal Welfare Act, the mini-pig study was approved by the animal ethics committee in Stockholm (N209/15). One cornea each of eight Göttingen mini-pigs was subjected to an alkali burn, while the contralateral untreated corneas served as controls. Alkali burns caused swelling of the ocular surface and lids, tearing for 2 weeks, squinting for up to 1 month, and corneal opacity (Figs. 3a, b, 4a). At 15 weeks post-burn, inflammation had resolved but the corneal haze remained (Figs. 3c, d, 4a, Supplementary Table 2). There were no changes to the pigs’ body weights and overall health status due to the burns or implantation of CLP-PEG-MPC and CLP-PEG hydrogels.

All cell-free implants epithelialized by the 7-week post-operative examination when the sutures used to stabilize the implants were removed. Between 7 weeks and 3 months post-operation, corneal haze increased (Fig. 3e–h, Fig. 4a, Supplementary Table 2) as stromal cells began migrating into the implants as visualized by in vivo confocal microscopy (IVCM). The Cochet–Bonnet aesthesiometer measures the pressure needed to produce a blink response by progressive shortening of a retractable nylon monofilament. Aesthesiometry showed decreased touch sensitivity that was most prominent at the 7-week and 3-month follow-ups, correlating to the corneal nerves being damaged during the surgery (Fig. 4b, Supplementary Table 3).

From 3 to 6 months post-operation, corneal haze decreased (Figs. 3g–j, 4a, Supplementary Table 2) while touch sensitivity increased as the newly remodeled areas became re-innervated (Fig. 4b, Supplementary Table 3). At 9 and 12 months post-operation, corneal haze was reduced to a light haze with a clinical score of 1 (Figs. 3k, l, 4a, Supplementary Table 2) while aesthesiometry showed touch sensitivity equivalent to that in unoperated corneas (Fig. 4b, Supplementary Table 3). IVCM confirmed the presence of a fully regenerated corneal epithelium (Fig. 3m, n), and regenerating sub-basal nerve plexus (Fig. 3p, q) and stroma (Fig. 3s, t) comparable to the controls (Fig. 3o, r, u). The endothelium, which was untouched during the surgery, remained intact (Fig. 3v, w) like the control (Fig. 3x).

The CLP-PEG-MPC implanted corneas maintained a thickness of 745 ± 83 µm (mean ± SEM) at 3 months post-op to 773 ± 60 µm at 12 months post-operation, comparable to that of unoperated contralateral corneas which were 789 ± 18 at 3 months and 801 ± 16 at 12 months (Fig. 4c, Supplementary Table 4). CLP-PEG only implanted corneas, however, showed a significant thickness increase of approximately 200 µm that was most pronounced at 3 months post-operation, persisting and remaining significant compared to the unoperated control (*p* = 0.0365) at 12 months post-operation.

Schirmer’s tear test showed that the alkali burn decreased tear production (Fig. 4d, Supplementary Table 5). However, tear production recovered and remained within normal values thereafter. Intraocular pressure was unaffected by the burn or hydrogel implantation, remaining within normal ranges of 8–20 mmHg over the entire study (Fig. 4e, Supplementary Table 6).

Implants were also grafted into the corneal stroma of a cat after ethical permission from the Maisonneuve-Rosemont Hospital Committee for Animal Protection and in accordance with the Association for Research in Vision and Ophthalmology Statement for the Use of Animals in Ophthalmic and Vision Research (Supplementary Note 1). The implants were grafted using limbal incisions at a depth of 350 µm (center of the stroma). Unlike the mini-pigs, the cat cornea was healthy and the implants served as inlays of 4 mm in diameter and 200 µm thickness. In the absence of alkali burns, both CLP-PEG-MPC and CLP-PEG implants remained optically transparent over the 14-month follow-up period (Supplementary Fig. 1, 2). Optical coherence tomography (OCT) showed the presence of clear implants at 14 months post-operation (Supplementary Fig. 3). The edges of the implants showed haziness on both OCT and slit-lamp imaging, which could be edge artefacts or cells that have migrated to the implants.

### Histopathology of implanted mini-pig corneas

Histopathology on the mini-pig eyes was performed by a certified, 3rd party veterinary pathologist (vivo Science GmbH, Gronau, Germany). The alkali burned corneal samples excised during implantation showed morphologically detectable corneal tissue damage characterized by focal epithelial erosion, multi-focal epithelial hyperplasia, and stromal hypercellularity together with dissociation and irregularity of the collagen fibers. The pathology observed was in accordance with established descriptions of post-burn healing.^33^

**Fig. 5:**
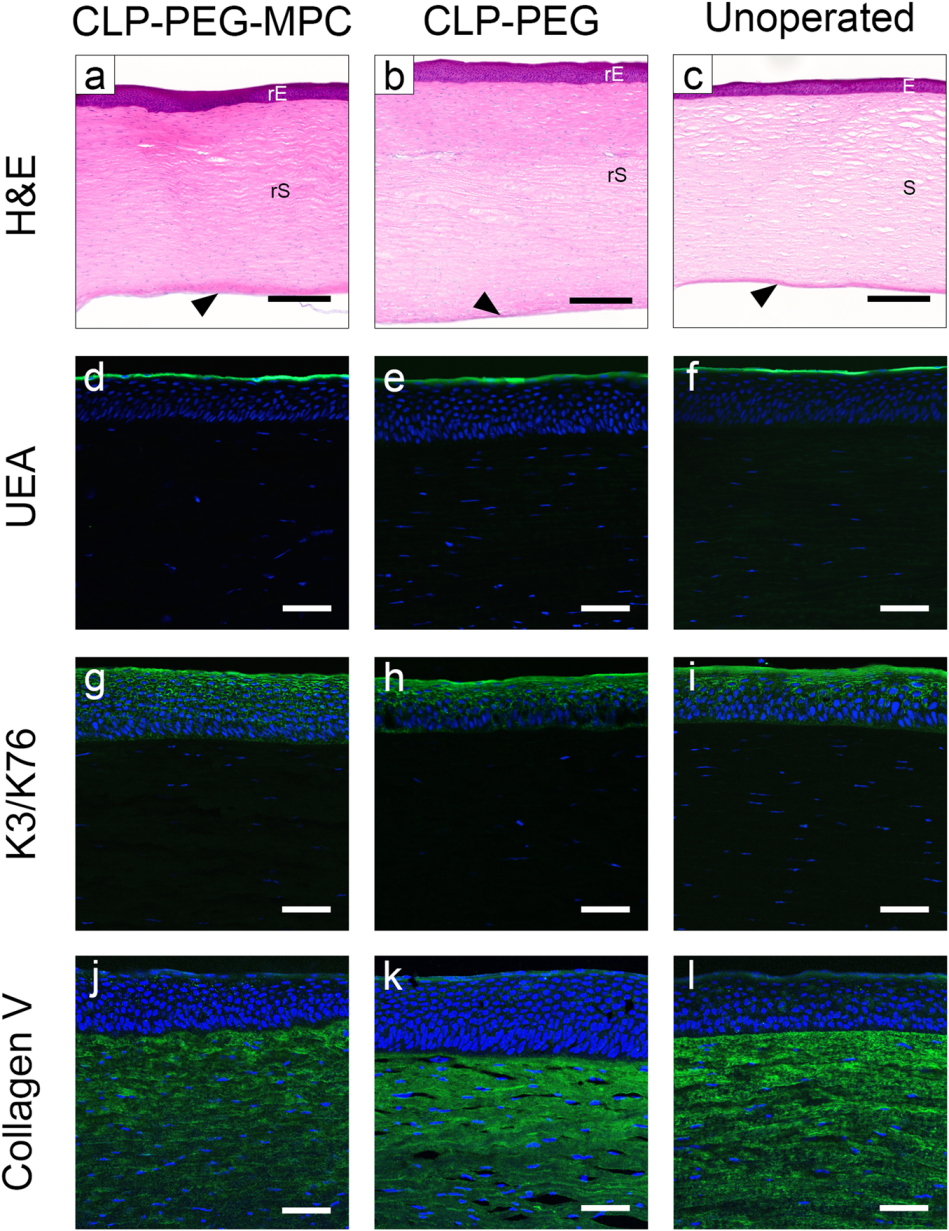
Regenerated mini-pig neo-corneas at 12 months after post-CLP-PEG-MPC implantation compared to CLP-PEG and controls. **a**–**c** Hematoxylin and eosin staining of the implanted corneas shows morphological similarity of the regenerated epithelium (rE) and stroma (rS) to the epithelium (E) and stroma (S) of an unoperated, healthy control cornea. The unoperated endothelium (arrowheads) remained intact in all samples. **d**–**f** Positive *Ulex Europaeus Agglutinin lectin* staining shows the presence of a tear film in both regenerated corneas and the control. **g**–**i** Terminally differentiated corneal cells were cytokeratin 3/76-positive in both regenerated neo-corneas. **j**–**l** Collagen V staining of the neo-cornea stromas shows the regeneration of corneal specific ECM components. Black scale bars, 500 µm; white scale bars, 200 µm.

**Fig. 6:**
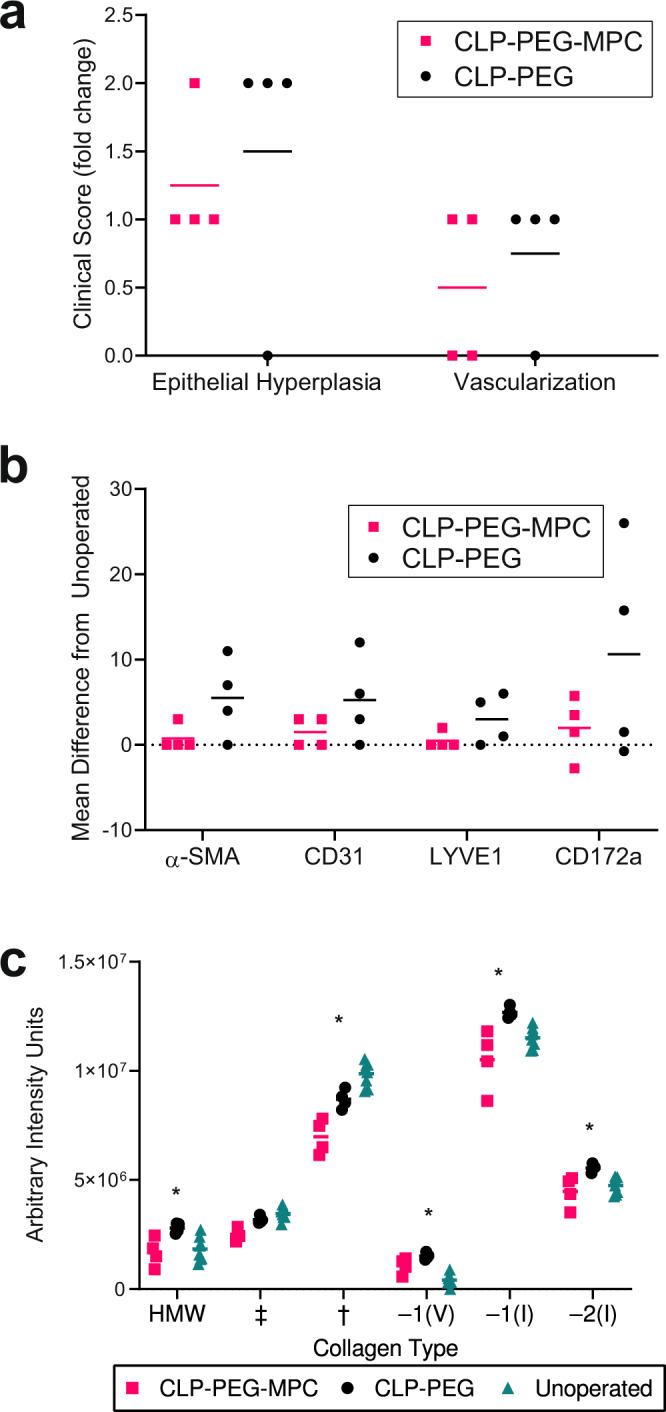
Histopathological, immunohistochemical and biochemical characteristics of regenerated neo-corneas at 12 months post-implantation with CLP-PEG-MPC implants compared to CLP-PEG implants. **a** Corneal epithelial hyperplasia was noticeably higher in the CLP-PEG only implants (black circles) compared to CLP-PEG-MPC implants (pink squares), while neovascularization was not markedly different. However, neither was statistically significant by the Mann–Whitney U test. **b** Mean cell counts normalized to the contralateral control eye for myofibroblast α-smooth muscle actin, blood vessel marker CD31, lymphatic vessel marker LYVE1, and the myeloid cell marker CD172a show no significant differences between CLP-PEG-MPC (pink squares) and CLP-PEG (black circles). Statistical analysis by unpaired, two-way t-test were performed with statistical significance set at *p*≤0.05. **c** Collagen content analysis of the central cornea in CLP-PEG-MPC (pink squares), CLP-PEG (black circles) and unoperated (aqua triangles) corneas. Statistical analysis of collagen by two-way ANOVA with Tukey’s multiple comparisons test. Data are displayed using the mean. *Unoperated vs CLP-PEG, *p*<0.05. †Unoperated vs. CLP-PEG-MPC, *p*≤0.05. ‡ CLP-PEG vs. CLP-PEG-MPC, *p*≤0.05. For all charts the unit of analysis is the eye: CLP-PEG *n*=4, CLP-PEG-MPC *n*=4, unoperated *n*=8.

At 12 months post-grafting, both regenerated CLP-PEG and CLP-PEG-MPC implanted corneas resembled healthy unoperated corneas (Fig. 5a–c, Fig. 6a). Although hyperplasia was more noticeable in CLP-PEG regenerated corneas than CLP-PEG-MPC, there were no significant difference (*p*=0.981, Supplementary Table 7). There was no difference in neovascularization either (*p*=1.000). Descemet’s membrane, which delineates the stroma from the underlying endothelium was intact, showing that neither the burns nor implants extended through to the endothelial compartment.

### Immunohistochemistry on regenerated mini-pig neo-corneas

Immunohistochemistry showed the regeneration of a tear film (Fig. 5d–f), terminally differentiated corneal epithelium (Fig. 5g–i), and cornea-specific collagen V in the stroma (Fig. 5j–l). Immunohistochemical staining for α-smooth muscle actin and CD31 as markers of blood vessels, lymphatic vessel endothelial hyaluronan receptor 1 (LYVE1) as markers of lymphatic vessels and CD172a as a marker of myeloid cells showed a trend towards higher expression in CLP-PEG implanted corneas (Fig. 6b, Supplementary Fig. 4, Supplementary Table 8). Expression of cathelicidin peptide LL37, a host-defence peptide, showed no marked differences (Supplementary Fig. 5).

### Biochemical analyses on regenerated mini-pig neo-corneas

Proteins extracted from regenerated neo-corneas contained significant levels of type I and type V collagens, the main corneal collagens (Fig. 6c). These results suggest that host cells had synthesized a collagenous matrix enriched in the same collagen types as are found in the normal cornea. Overall, there were differences in the amounts of individual α chains, β-dimers and γ trimers of collagen between CLP-PEG-MPC and CLP-PEG only implants. CLP-PEG-MPC has equivalent amounts of covalently crosslinked high molecular weight collagen to unoperated corneas, but showed decreased amounts of individual α chains, β-dimers and γ trimers compared to the CLP-PEG and unoperated groups. CLP-PEG implants had significantly less cornea-specific type V (α1(V)) collagen than CLP-PEG-MPC or untreated corneas (Fig. 6c, Supplementary Table 9).

### Electron microscopy on regenerated mini-pig neo-corneas

**Fig. 7:**
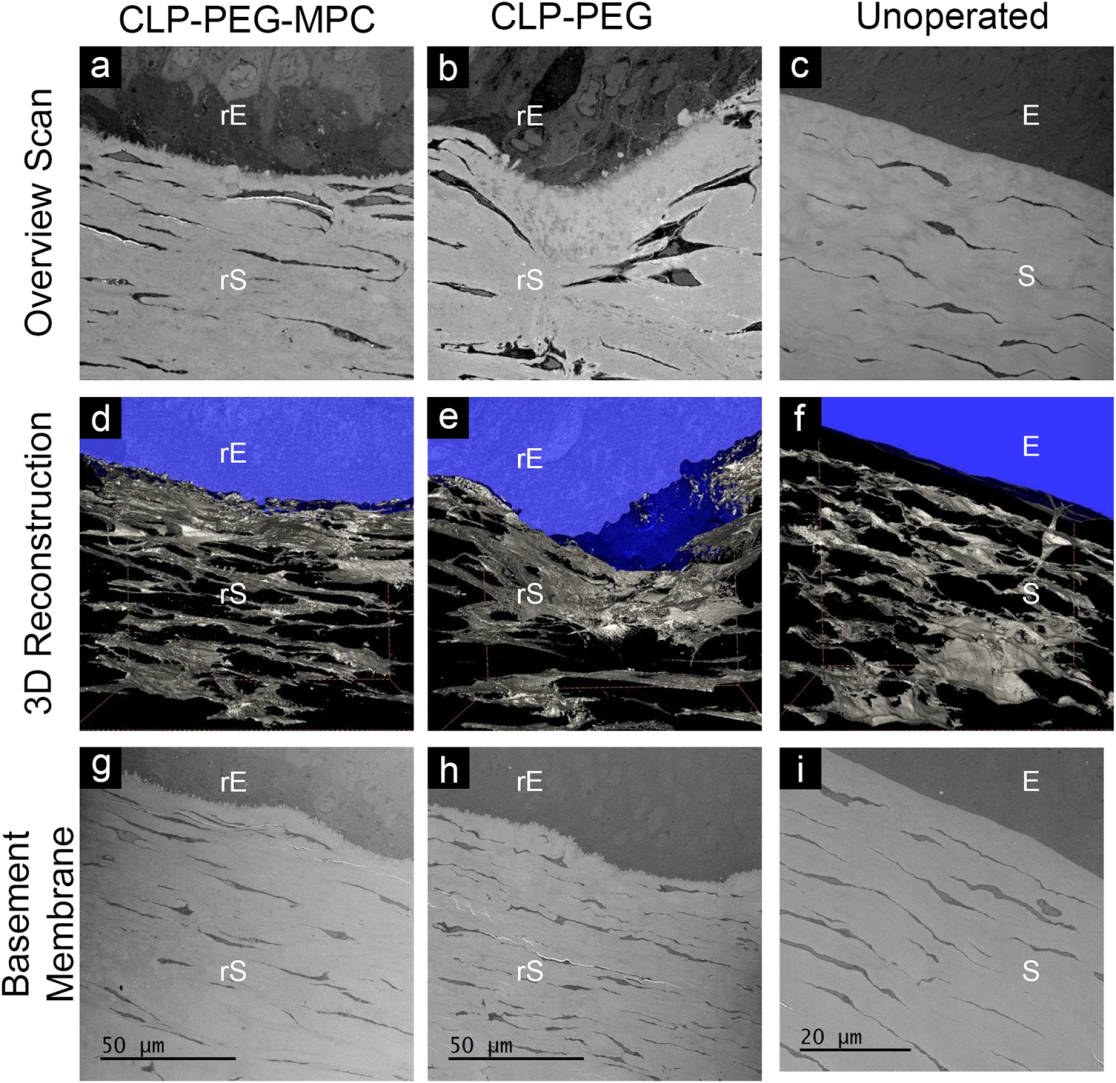
Electron micrographs of regenerated neo-corneas after CLP-PEG and CLP-PEG-MPC implantation. Serial block face scanning electron microscope overview scans show comparable epithelial and stromal compartments (**a**–**c**). 3D reconstructions (**d**–**f**) of the neo-corneas after digital removal of the extracellular matrix show that keratocytes within the stroma are arranged in interconnected lamellae. The lamellae in the CLP-PEG only implanted corneas are less organized than in the other two groups. **g**–**i** Transmission electron microscopy of the corneas indicates that both operated groups (**g**, **h**) have invaginated basal epithelia. E, epithelium; S, stroma; r, regenerated.

Serial block face scanning electron microscopy (SBF-SEM) (Fig. 7a–f) and transmission electron microscopy (Fig. 7g–i) showed that both neo-corneas resembled naïve corneas, except that the basal aspect of their basal epithelial cells showed numerous invaginations. SBF-SEM also revealed a more regular lamellar keratocyte arrangement in CLP-PEG-MPC neo-corneas (Fig. 7a, d) resembling that of the healthy, unoperated controls (Fig. 7c, f). However, the CLP-PEG neo-corneas had more unevenly spaced stromal keratocytes with fewer cell layers (Fig. 7b, e), suggesting a still-evolving morphology.

### Extracellular vesicles and exosomes

**Fig. 8:**
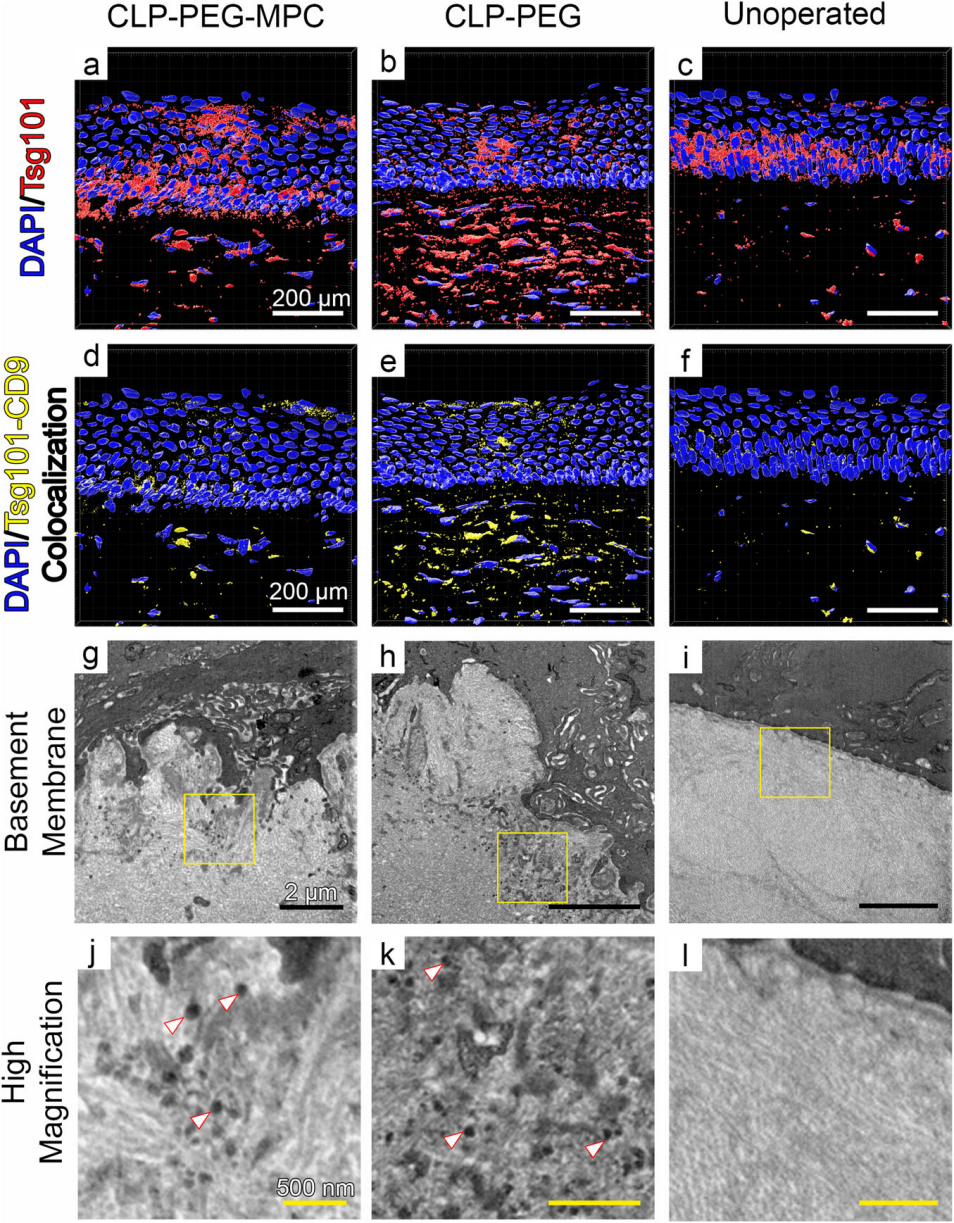
Extracellular vesicles and exosome secretion by regenerating CLP-PEG-MPC and CLP-PEG implanted corneas. **a**–**c** Greater amounts of Tsg101+ EVs (red staining) were present in the grafted regions, with the highest level of secretion in the CLP-PEG implants compared to CLP-PEG-MPC implants and unoperated controls. DAPI (blue staining) was used to identify the cell nuclei. **d**–**f** CLP-PEG implants also had the highest amount of Tsg101+, CD9+ exosomes represented by yellow staining. White scale bars, 200 µm. **g**–**i** High resolution TEM of the basement membrane confirms the increased release of exosomes from the epithelium into the stroma. Black scale bars, 2 µm. **j**–**l** High magnification of the inset yellow-boxed areas showing the presence of exosomes (white arrowheads) in the stroma. Yellow scale bars, 500 nm.

Intense staining for Tsg101-positive extracellular vesicles (EVs) was seen in CLP-PEG neo-corneas (Fig. 8b) compared to unoperated controls (Fig. 8c). Colocalization of Tsg101 and the exosomal surface marker CD9, showed that many of the EVs were exosomes (Fig. 8e). The CLP-PEG-MPC implants showed a slightly higher amount of both EVs (Fig. 8a) and exosomes (Fig. 8d) compared to the unoperated control corneas (Fig. 8c, f). TEM of the corneal basement membrane showed the presence of exosomes in both grafted materials (Fig. 8g, h, j, k) but observably more copious amounts in the CLP-PEG neo-corneas.

## Discussion

Our aim was to develop a fully synthetic peptide-based corneal implant with inflammation-suppressing properties. We showed that CLP-PEG-MPC implants, like previously described CLP-PEG ones, were readily and reliably mouldable.^29^ Both implants had refractive indices of 1.34, in keeping with their high water content. However, while both implants were highly transparent in visible light, CLP-PEG-MPC but not CLP-PEG implants filtered up to 60% of potentially damaging UV-A, which is essential for lens and retina protection (from cataract and macular degeneration). CLP-PEG hydrogels were stiffer and less elastic than those containing MPC. Interestingly, a similar trend was previously observed upon incorporation of MPC within RHCIII implants.^39^ Although the CLP-PEG-MPC implants had lower overall tensile strength, their elasticity made them sufficiently robust for surgical handling and implantation with overlying sutures. Neither hydrogel was as tough as the human cornea, but they had properties close to those of RHCIII corneal implants successfully tested in a clinical trial for over four years.^18,19^ Here, as in Fagerholm et al.,^19^ the original implant was remodeled during corneal regeneration. Furthermore, Jangamreddy et al.^29^ showed that the weaker, cell-free CLP-PEG hydrogels implanted into rabbit corneas transformed into regenerated neo-corneas with mechanical properties approximating those of healthy, unoperated corneas, the desired end-points. Here, the objective was to synthesize a decreased-cost, peptide-based implant with the immunosuppressive qualities of MPC.

Both hydrogels supported HCEC proliferation in vitro, with the slower initial growth on the MPC-containing gels attributed to the non-adherent “slippery” nature of phosphorylcholine-derived hydrogel surfaces.^34,35^ When evaluating the effect of crosslinking agents on the activation of dendritic cells, EDC-NHS was cytotoxic and killed the cells at levels where DMTMM yielded live cells with minimal activation compared to positive LPS controls. These observations correlate with Samarawickrama et al.^31^, who showed that 0.5% (w/v) EDC-NHS was more toxic than 1% (w/v) DMTMM. The components and prepared CLP-PEG and CLP-PEG-MPC hydrogels had significantly lower capacity to activate dendritic cells compared to LPS. Hence, the hydrogels would be well-tolerated as implants.

The alkali burn cornea model is a well-established ophthalmology model of severe pathology in rodents and rabbits, resulting in marked inflammation and often neovascularization. In a study by Rehany and Waissman, 20 rabbits with alkali burns required intramuscular injections of 25 mg/kg cyclosporin A daily for 30 days post-operation to allow allograft tolerance.^36^ Rabbits that did not receive an intensive steroid regimen uniformly rejected the allografts following severe vascularization. Here, we adapted the alkali burn model to Gottingen mini-pig corneas. A certified veterinary pathologist confirmed the resulting burn pathology of stromal disruption and hypercellularity in the excised scarred corneal tissue.^33^

Both CLP-PEG-MPC and CLP-PEG implants showed healing and regeneration over the 12-month post-operation period, in the absence of steroids or immunosuppressive drugs. The presence of terminally differentiated epithelial cells, regenerated tear film and host-defence peptide, LL37, showed that both hydrogels promoted functional epithelial regeneration. However, CLP-PEG-MPC implants had significantly reduced corneal epithelial hyperplasia and stromal thickening, supporting the contention that MPC suppresses corneal inflammation. While not apparent in H&E histopathology sections, immunohistochemistry revealed that CLP-PEG-MPC implanted corneas also showed a trend towards reduced blood and lymphatic vessels and fewer myeloid cells in the graft site. The phosphorylcholine network appeared to decrease corneal haze and improve the rate of nerve regeneration to restore the corneal blink response. Unfortunately, although CLP-PEG-MPC showed a trend towards improved performance, the small number of pigs in each experimental group used due to cost constraints of performing a certified GLP study in a large animal model, did not allow sufficient power to discriminate and show statistical significance.

CLP-PEG-MPC corneas expressed lower amounts of monomeric (α), dimeric (β), and trimeric (γ) collagen than CLP-PEG implanted ones, although they expressed similar amounts of crosslinked collagen fibrils (HMW) to unoperated controls. More interestingly, the CLP-PEG-MPC implanted corneas had higher amounts of type V collagen, which is a collagen that is present in the corneas in enhanced amounts and shown to be involved in the maintenance of corneal transparency.^37^ CLP-PEG-MPC implants also had a lower amount of EVs and exosomes than CLP-PEG implanted corneas. CLP-PEG has previously been shown to stimulate regenerating corneal cells to produce large amounts of cornea-specific type V collagen associated exosomes.^29^ Taken together, these results strongly suggest that regenerated CLP-PEG-MPC neo-corneas were at a more advanced stage of corneal regeneration than the CLP-PEG ones, with more type V collagen in its extracellular matrix, possibly due to the modulation of inflammation in the former.

The CLP-PEG biomaterial in the alkali burned corneas resulted in low-grade haze throughout the 12-month follow-up period with blood vessels in three of four pigs. Incorporation of MPC decreased the haze in the regenerated neo-tissues but they did not reach full optical clarity. There were also residual traces of neovascularisation. However, when CLP-PEG-MPC and CLP-PEG hydrogels were implanted within the stroma of a healthy, non-burned cat cornea, both implants remained optically transparent over 14 months. Both hydrogels performed equivalently as low-risk implants, with the presence of MPC only noticeable under high-risk grafting conditions.

The CLP-PEG-MPC implants were therefore able to restore the alkali burned corneal environment to one that resembled an uninflamed state, allowing regeneration of corneal epithelium, stroma and nerves. The presence of MPC circumvented the increased thickness seen in the non-MPC-containing implants. These results confirm the decreased neovascularization observed in rabbits^38^ and the stable regeneration of six high-risk patients with active ulcers and scarring in a clinical trial using full-length RHCIII-MPC implants.^21^ Combined, these studies show that MPC is an effective inflammation-suppressing polymer that also imparts elasticity to the overall hydrogel. The novelty of the present study is that CLP-PEG-MPC implants combine inflammation suppression with a fully synthetic collagen analog comprising CLP and PEG. The short, synthetically produced CLPs are readily manufactured without the need for co-expression of several full-length proteins needed to produce recombinant human collagen and avoids possible xenogeneic-origin allergy^39^ or zoonotic transmission of pathogens such as viruses that may result from animal-derived materials.^40^ Furthermore, the use of synthetic analogs allows for future modification and customization of implants for personalized medicine, which is difficult with more chemically inert full-length collagens. The results indicate that evaluation of CLP-PEG-MPC implants in a clinical trial is merited.

With a formidable 12.7 million patients on waiting lists worldwide for corneal transplantation and a severe shortage of human donor corneas, CLP-PEG-MPC implants may in the near future be an alternative treatment, and further address the unmet need of patients with inflammation and severe conditions who are not amenable to standard donor corneal tissue transplantation.

## Methods

### Fabrication

CLP (CG(PKG)_4_(POG)_4_(DOG)_4_) (AmbioPharm, SC, USA) was conjugated to an 8-arm poly(ethylene glycol) with a hexaglycerol core (Sinopeg Biotech Co. Ltd., Beijing, China).^28^ In brief, CLP was conjugated to PEG at pH 4.5, sterile filtered and dialyzed using a 12–14 kDa membrane to remove unreacted CLP. The product was lyophilized and re-dissolved at 12% (w/w). CLP-PEG hydrogels were produced using 4-(4,6-dimethoxy-1,3,5-triazin-2-yl)-4-methylmorpholinium chloride (DMTMM) at molar ratio of 1:2 CLP-PEG:DMTMM. CLP-PEG-PC implants were manufactured using an additional phosphorylcholine network based on the interpenetrating network from our previous recombinant human collagen type III-phosphorylcholine implants.^21^ The phosphorylcholine network is composed of 2-methacryloyloxyethyl phosphorylcholine (MPC) (Paramount Fine Chemicals, Beijing, China) and polyethylene glycol diacrylate (PEGDA) (Sigma-Aldrich, St. Louis, MO) The ratio of CLP-PEG:MPC was 2:1 (w/w) and MPC:PEGDA was 3:1 (w/w). Ammonium persulfate and *N,N,N*′*N’*′-tetramethylethylenediamine (Sigma-Aldrich, St. Louis, MO) were both used as polymerization initiators at a ratio of MPC:APS of 1:0.03 and APS:TEMED of 1:0.77. Corneal implants were cast as 10 mm diameter, 500 µm thick curved hydrogel molds (custom manufactured by Formteknik, Anderstorp, Sweden). Dogbone-shaped hydrogels of the same thickness were used for physical, mechanical and in vitro testing.

The CLP-PEG-MPC and CLP-PEG hydrogel implants used in the in vivo mini-pig study and supplemental cat study were custom manufactured by UAB Ferentis (Vilnius, Lithuania) and stored in PBS containing 1% chloroform.

### NMR spectroscopy

Incorporation of MPC into the CLP-PEG-MPC hydrogels was assessed with ^13^C and ^31^P Cross Polarization Magic-Angle Spinning (CPMAS) nuclear magnetic resonance (NMR) spectroscopy on a Bruker AVANCE 400 MHz spectrometer at room temperature. CLP-PEG only hydrogels served as negative controls. ^13^C NMR produced convoluted spectra in the region between 55 and 70 ppm that made it difficult to interpret, but ^31^P NMR gave definitive peaks. Peak analysis was performed using Mnova (Mestrelab Research, Santiago de Compostela, Spain).

### Young’s modulus and mechanical strength

Dogbone-shaped hydrogels (500 μm thick) were evaluated using an Instron electromechanical universal tester (Model 3342, Instron, Norwood, MA) equipped with Series IX/S software. The hydrogels were washed in 1X PBS for 1 h before testing and blotted to remove excess water. The elongation tests were performed using a crosshead speed of 10 mm min^‒1^.

### Rheology

Circular hydrogels, 8 mm in diameter and 500 µm thick, were tested using a Discovery Hybrid-2 rheometer (TA Instruments, New Castle, DE, USA) fitted with an 8 mm circular geometry. After blotting to remove excess water, the hydrogels were compressed to an approximate pressure of 1 N of axial force. An amplitude sweep was run from 0.1 to 500% using an angular frequency of 10 rad/s. Analysis was conducted using Trios v5.1.1.46572 (TA Instruments, New Castle, DE, USA).

### Light transmission of hydrogels

Flat hydrogel sheets of 500 µm thickness and 5 mm × 10 mm dimensions were evaluated for light transmission between 250 nm and 800 nm. Each hydrogel was placed on the inside wall of a quartz cuvette filled with PBS. The absorption was read using a Spectramax M2e series plate/cuvette spectrophotometer (Molecular Devices, San Jose, CA, USA). A cuvette filled with PBS was used as the baseline reference. The percent transmission was calculated from the measured absorbances.

### Refractive index

The refractive index of the hydrogels was measured at RT on an Abbemat 300 (Anton Parr) refractometer.

### Water content of hydrogels

A baseline measurement of a blotted hydrogel was obtained as the starting “wet weight” (W_0_) of the hydrogel. The hydrogels were then dried until a stable “dry weight” (*W*) was obtained. The percent water content of the hydrogels (W_t_) was calculated according to the equation: *W*_t_ % = (*W*−*W*_0_)/*W* %.

### Collagenase degradation

Samples were equilibrated in Tris-HCl buffer (0.1 M, pH 7.4) overnight. Hydrogels were then incubated in 5 mL 5 U/mL type I bacterial collagenase dissolved in Tris-HCl at 37 °C. The undigested mass was weighed at time 0 (*W*′_0_) and every 8 h (*W*′_t_) for 2 days (48 h). At every interval, surface water was blotted away, and samples were weighed using an ultra-microbalance (SE2, Sartorius, Göttingen, Germany). At every weighing occasion, the collagenase solution was replaced with a fresh collagenase mixture. The percentage of mass remaining after digestion was calculated according following equation: Residual mass (%) = (*W*′_t_/*W*′_0_) × 100%.

### Permeability

Permeability studies were conducted for two concentrations of CLP-PEG hydrogels (12% and 8%) and one concentration of CLP-PEG-MPC hydrogel (9%).^41^ Hydrogels or human amniotic membrane (hAM) were clamped in an Ussing Chamber system (Physiologic instruments, San Diego, CA) with P2300 EasyMount Diffusion Chambers and P2307 sliders. A 700 Da Alexa Fluor^®^ 568 hydrazide sodium salt (10 μM) was placed in aqueous solution in the donor chamber and PBS was placed in the recipient chamber. Samples were drawn at multiple time points and fluorescence was quantified using 590 nm excitation wavelength and a 642 nm emission wavelength by a Wallac Viktor2 1420 Multilabel counter (PerkinElmer, Waltham, MA, USA). The apparent permeability coefficient (*P*_app_, cm s^−1^) was calculated using the equation *P*_app_ = (d*C*/d*t*)/(60*C*_0_*A*) [d*C*/d*t* is the slope, *C*_0_ the initial concentration of the donor chamber and A the exposed surface area of the sample in the slider (0.031 cm^2^)]. Cumulative permeability was calculated as the percentage of diffused fluorescent marker from the donor chamber to the receiving chamber during the experiment.

### Human corneal epithelial cell culture

A stable GFP-HCEC cell line was established by the transfection of SV40 immortalized HCEC cells (Gift of H Handa, Division of Ophthalmology, Kinki Central Hospital, Hyogo, Japan) with a vector containing a puromycin-resistant gene together with GFP, using the Lipofectamine 2000 Transfection Reagent (Life Technologies, California, USA).^42,43^ Selection of puromycin-resistant cells with 2 μg ml^–1^ of puromycin added to the medium was performed to obtain stable GFP-expressing lines. The initial immortalized HCEC line was characterized using the expression of keratin and large T antigen.^42^ GFP-HCECs were subsequently characterized by morphology, and expression of Integrin β1 and focal adhesion kinase cell proliferation rate.^43^ These cells were not checked for mycoplasma contamination.

To conduct proliferation studies, CLP-PEG and CLP-PEG-MPC hydrogels were punched using a 5 mm biopsy punch and placed in a 96 well plate. GFP-HCECs were seeded into the control wells and onto the materials at a density of 5000 cells/well. GFP-HCECs were supplemented with keratinocyte serum-free medium (KSFM; Gibco, ThermoFisher, Waltham, MA, USA) containing 0.05 mg/mL bovine pituitary extract, 5 ng/mL epidermal growth factor, and 1 mg/mL penicillin/streptomycin and their growth monitored for 7 days in a humidified incubator at 37 °C and 5% CO_2_. Growth was assessed by photographing the cultured cells and examining the % coverage of the culture dishes.

For the Alamar Blue and live dead studies, the hydrogels were cut into 6-mm-diameter and overnight immersed in the cell culture media. 5,000 HCECs were seeded on top of each hydrogel for culturing with keratinocyte serum-free medium (KSFM) supplemented with 50 μg/ml bovine pituitary extract and 5 ng/ml epidermal growth factor (EGF) (Gibco, California, USA) in a 37 °C and 5% CO_2_ incubator. Media was changed every alternative day. Cells seeded on tissue culture plate (TCP) were used as control. The Alamar Blue study was performed at day 1, day 4 and day 6 after cell seeding.^44^ At each time point, resazurin sodium salt was added to the cell culture wells to obtain the final concentration of 0.004% (w/v) and incubated for 3 h. Afterwards, the media was transfer to a new 96 well plate and read on a BioTek plate reader (Synergy 2, BioTek Instruments; Winooski, VT) at 530/25 nm for excitation and 590/35 nm for emission. At day 6, live/dead staining was performed with a staining kit (Life Technologies Corporation, Oregon, USA), where cells were double-stained by calcein acetoxymethyl (Calcein AM) and ethidium homodimer-1 (EthD-1). Images were taken by using a fluorescence microscope (Zeiss Axio Observer Z1, Carl Zeiss Microimaging GmbH, Jena, Germany).

### Bone marrow-derived dendritic cell (BMDC) activation assay

Bone marrow was isolated from the femur and tibia of 6- to 12-week-old male C57BL/6J mice (*Mus musculus*). Cells (1×10^6^/well) were seeded onto 6-well suspension culture plates in RPMI 1640 containing 10% (v/v) fetal bovine serum (Wisent, Saint-Jean-Baptiste, QC), 0.5 mg/mL penicillin-streptomycin-glutamine, 10 mM HEPES, 1 mM sodium pyruvate, 55 μm β-mercaptoethanol and 2.5 ng/mL granulocyte-macrophage colony-stimulating factor (GM-CSF) (Gibco, Waltham, MA). Complete RPMI, containing 5.0 ng/mL GM-CSF, was exchanged for half of the media on days 2 and 3 of culture. Cultures were maintained for 6 days, then collected and subject to density gradient centrifugation using Histodenz™ (Sigma-Aldrich, St. Louis, MO) to separate the enlarged BMDCs. The selected cells were seeded at a density of 1x10^6^ cells/well on a 24 well plate for materials testing.

Hydrogel components (EDC/NHS, DMTMM, CLP, CLP-PEG, and MPC) were applied to the BMDCs at an equivalent total mass to a 10 mm, 500 μm thick hydrogel disk, to simulate the total amount present in a complete corneal implant. Hydrogels disks (6 mm diameter, 500 μm thick) were incubated with BMDCs for 24 h. Lipopolysaccharide (LPS) was used as a positive control. BMDCs were collected and labeled with direct-conjugate antibodies for CD11c, CD40, CD80 and CD86 (Supplementary Table 10) and Zombie Aqua™ Fixable Viability Kit (BioLegend, San Diego, CA). Flow cytometry was performed using a BD LSR II flow cytometer using FACSDiva as collection software and analyzed using FlowJo software (Becton, Dickinson & Company). The cells were gated for size and granularity using a FSC/SSC gate (Supplementary Fig. 6a), followed by a gate to remove dead cells, based on low Zombie-Aqua (Supplementary Fig. 6b). The live cells were gated for CD11c high, autofluorescence low (Supplementary Fig. 6c) and this is the gate that was subject to subsequent analysis. Mature dendritic cells composed 50–99% of the live gate, as there was significant cell death in BMDCs exposed to the toxic crosslinker EDC-NHS.

### In vivo study in Göttingen mini-pigs

A study to evaluate the safety and biocompatibility of CLP-PEG-MPC and CLP-PEG implants in mini-pigs was performed in compliance with the Swedish Animal Welfare Ordinance and the Animal Welfare Act, with ethical permission from the local ethical committee in Stockholm (N209/15), and in accordance with OECD Principles of Good Laboratory Practices (GLP), ENV/MC/CHEM (98) 17, 1997, by Adlego Biomedical AB (Stockholm, Sweden). All animals were examined prior to alkali burn, and after alkali burn prior to surgical implantation of CLP-PEG or CLP-PEG-MPC hydrogels. The pig samples size is based on the standard amount for safety and toxicology testing (*n*=4 per group). The pigs were randomly allocated to the two biomaterials groups by the veterinary team at Adlego AB (Solna, Sweden). The corneal surgeons were blinded as to which of the CLP-PEG or CLP-PEG-MPC implants were implanted in each pig cornea. No data were excluded from this study and this study has not been replicated. The full GLP study report by Adlego AB is available on Dryad.^45^

Eight female Göttingen mini-pigs (*Sus scrofa domesticus*) were placed under general anesthesia and pre-treated with tetracaine 1% eye drops (Chauvin Pharmaceuticals Ltd, UK). Alkali burns were created in the right corneas of each pig. A 5 mm, circular piece of filter paper soaked in 1M NaOH was placed on the right cornea of each animal for 60 seconds, followed by a thorough rinse in 0.9% saline, by flooding the treated cornea to remove excess NaOH. Buprenorphine (0.05 mg/kg, Vetergesic, Orion Parma, Finland) was administered at the end of the procedure and when signs of pain and discomfort were observed during recovery. The injured eyes received chloramphenicol eye drops (5 mg/mL, Santen), twice daily for 8 days post-procedure. The alkali burns were evaluated by slit lamp under sedation, 6 weeks after the procedure. A full clinical exam was performed at 13 weeks, immediately prior to surgery.

Each alkali burned cornea underwent an anterior lamellar keratoplasty (ALK) procedure to replace the anterior 2/3 s of each scarred cornea. Under general anesthesia, each alkali burned cornea (average thickness 750 µm) was trephined to a depth of 500 μm using a 6.5 mm diameter Barron Hessberg trephine. A corneal diamond knife was used to complete the lamellar dissection. A 500 μm thick CLP-PEG-MPC or CLP-PEG only implant, purchased from UAB Ferentis (Vilnius, LT) was trephined to a diameter of 6.75 mm, placed in the wound bed and sutured in place using 10-0 nylon (MANI Ophthalmic, Tochigi, Japan). Six interrupted sutures were used to retain the implants where possible; otherwise overlying mattress sutures were used. Each operated eye received a single dose of 3 mg/mL dexamethasone and 1 mg/mL tobramycin eye drops (Tobrasone, Alcon, Sweden) at the end of surgery.

After surgery, the animals were examined daily for 10 days and then weekly. The operated eye received one drop of Tobrasone three times daily for 5 weeks. Suture removal was performed under general anesthesia at post-operative 7 weeks. Full eye examinations under general anesthesia were performed at 7 weeks as well as 3, 6, 9, and 12 months post-operation. Restrained, fully conscious pigs were assessed for central corneal touch sensitivity in using a Cochet-Bonnet aesthesiometer (Handaya Co, Tokyo, Japan). After topical anesthesia and confirmation of the extinction of the corneal blink response, the eyes were also tested for tear production using Schirmer’s tear test (TearFlo, Hub Pharmaceuticals USA), under sedation and prior to general anesthesia. For the remaining examinations, animals were examined under general anesthesia. Examinations include measurements of intraocular pressure (TonoVet Tonometer, Icare Finland Oy, Finland), and pachymetry (Handy Pachymeter SP-100, Tomey, AZ, USA). A full slit lamp evaluation (Kowa SL-15 Portable Slot Lamp, Kowa Company, Ltd., Aichi, Japan) was conducted in the presence of fluorescein eye drops (Lidocaine-fluorescein 4% + 0.25%, Chauvin Pharmaceuticals Ltd.) using the McDonald-Shadduck scoring system to quantify anterior segment findings. Each eye was scored from 0 to +4 on conjunctiva congestion, swelling and discharge, aqueous flare, iris involvement, and percent corneal haze. Full thickness IVCM was performed at peripheral and central portions of the implant and similar positions in the unoperated corneas (Heidelberg HRT3 with Rostock Cornea Module with HEYEX software, Heidelberg, Germany).

The pigs were euthanized at 12 months post-operation with an overdose of pentobarbitol (100 mg/mL, Allfatal Vet, Omnidea, Sweden) and corneas were dissected out with a 2 mm rim of surrounding conjunctiva. The center 2 mm of each cornea was excised with a biopsy punch and snap frozen in isopentane chilled with dry ice for collagen content analysis. The remaining cornea was then quartered for further processing.

### Histopathology of mini-pig corneas

One quarter of the cornea was fixed in 4% paraformaldehyde in phosphate buffer for Haematoxylin and Eosin (H&E) staining and histopathological analysis under GLP by a certified veterinary pathologist at in vivo Science GmbH (Gronau, Germany). Standard H&E stained research sections were imaged using a Zeiss AxioObserver Z1 inverted light microscope using the tiling function in Zen Blue v2.3 at 20x magnification (Carl Zeiss Microscopy, Göttingen, Germany).

### Collagen analysis of mini-pig central cornea

Frozen biopsied corneal samples from within the surgical areas of both implanted and control corneas were thawed and re-suspended in 10 mM HCl at a wet weight to volume ratio of 1:35. Pepsin (Roche, catalog# 200911, lot#93100120) was added to a final concentration of 1 mg/ml from a 10 mg/ml stock solution prepared just before use. The samples were digested with pepsin at 2–8 °C for 96 h and the soluble fraction was recovered by centrifugation in a microfuge at 16,000*g* for 30 min at 2–4 °C. An aliquot of the pepsin soluble fraction was mixed with NuPAGE 4X LDS sample buffer (Life Technologies) denatured at 75  °C for 8 min and analyzed on 3–8% Tris-acetate gels under non-reducing conditions. Proteins were visualized by staining with Gelcode Blue (Pierce). Prestained broad range marker (New England Biolabs, catalog# P7712) and porcine skin type I collagen (Koken Co. Ltd., Japan) were used as molecular weight standards. To quantitate the amounts of type I and type V collagens in control and operated corneas, densitometric scans of the stained gels were made to obtain relative numerical units using a GE Healthcare Image Quant 350. ANOVA was performed to determine statistical differences using GraphPad Prism 5 on a DELL Latitude E6420 computer using Windows 7 OS.

### Immunohistochemistry of mini-pig corneas

One quarter of each cornea was fixed in 4% paraformaldehyde in phosphate buffer containing 5% sucrose. The corneas were processed through a sucrose gradient prior to embedding in OCT. Immunohistochemical staining was conducted using antibodies against cytokeratin, CD172a, smooth muscle actin, LYVE-1, CD31, collagen V (Supplementary Table 11). Corneal sections (7 μm) were fixed in cold 4% PFA followed by ice-cold methanol, air-dried, washed in PBS and then blocked with 5% normal goat serum (NGS) in PBS. All primary antibodies were incubated overnight at 4 °C. Slides were washed in PBS with 1% Tween 20 and then incubated with secondary antibodies diluted 1:1000 in blocking solution. After washing the slides were dehydrated and mounted in Vectashield Antifade Mounting Medium with DAPI (Vector Laboratories, Burlingame, CA, USA).

Slides stained using lectin were washed in PBS, stained with lectin overnight at 4 °C, washed and counterstained with DAPI, before mounting in Vectashield Antifade Mounting Medium. Fluorescent images were obtained with a confocal laser-scanning microscope (LSM800, Carl Zeiss Microscopy, Göttingen, Germany).

Immunohistochemical staining of exosomes was conducted using dual staining with CD9 and TSG101, per the ISEV positive staining requirement for one transmembrane protein and one cytosolic protein associated with exosomes.^46^ Corneal sections were air dried, washed in PBS, and permeabilized in PBS with 0.3% Triton-X. The sections were washed and incubated in Tris-buffered saline (TBS) containing 50 mM ammonium chloride. The samples were blocked in PBS with 5% NGS and 0.01 g/mL saponin at room temperature prior to incubation in primary antibodies overnight. Slides were washed in PBS containing 5% FBS and 0.01 g/mL saponin and incubated in secondary antibodies diluted in blocking at room temperature. The samples were quenched for autofluorescence using Vector® TrueVIEW™ Autofluorescence Quenching Kit (Vector Laboratories, Burlingame, CA, USA). Slides were stained with DAPI (5 μg/mL) for 10 min prior to mounting in Vectashield Antifade Mounting Medium (Vector Laboratories, Burlingame, CA, USA). Fluorescent images were obtained with a confocal laser-scanning microscope (LSM880, Carl Zeiss Microscopy, Göttingen, Germany). Images were denoised using Zen Blue v2.3. Three-dimensional reconstructions of the slices were generated in Imaris v9.2.1 (Bitplane Inc., Concord, MA, USA). Surfaces were reconstructed for Tsg101 using a manual threshold value of 5. A co-localization channel was constructed for CD9 and Tsg101 and surfaces were reconstructed using a manual threshold value of 1.5. All surfaces used a minimum voxel threshold of 10 and surface grain threshold of 0.141 μm.

Immunohistochemistry for LL37 was performed as described above for exosomes. Three-dimensional reconstructions of the slices were generated in Imaris v9.2.1 (Bitplane Inc., Concord, MA, USA). Ten 700 μm^3^ regions of interest (ROIs), five in the epithelium and five in the stroma, were generated for each slice. LL37 staining in each ROI was reconstructed as spots with an intensity threshold of 7.57 and a minimum voxel threshold of 10. The number of spots and sum intensity of each ROI was analyzed by the total volume of the ROIs. A one-way Kruskal–Wallis test was performed using a Dunn’s multiple comparisons test (GraphPad Prism v9.0.2, GraphPad Software, LLC., San Diego, CA, USA).

### Electron microscopy of mini-pig corneas

The mini-pig corneal/construct samples were fixed using 2.5% glutaraldehyde/2% paraformaldehyde in 100 mM cacodylate buffer pH 7.2 at room temperature (RT) for 12 h after dissection and placed in 100 mM cacodylate storage buffer pH 7.2. The samples were processed for TEM and SBF-SEM using a method for the generation of high backscatter electron contrast for serial block face scanning (SBF SEM).^47^ After the fixation, the sample quadrants were cut into thin <1 mm slices to preserve the positioning of the implant in relation to host cornea. Each sample slice was transferred to 1.5% potassium ferricyanide/1% osmium tetroxide in cacodylate buffer for 1 h and then washed in distilled water. The samples were then placed sequentially in 1% aqueous thiocarbohydrazide, 1% osmium tetroxide and 1% aqueous uranyl acetate, each for 1 h. All the staining steps were followed by 30 mins distilled water washing steps.

The samples were then incubated for 1 h in a solution of lead aspartate at 60 °C and then washed in two changes of distilled water for 30 min. They were dehydrated in an ethanol series from 70% through to 100% and, following via propylene oxide infiltration, they were embedded in CY212 (TAAB) epoxy resin and polymerized for 24 h at 60 °C.

The surfaces of polymerized resin blocks were then trimmed and attached to Gatan (PEP6590) specimen pins. The pins were gold coated and transferred to a Zeiss Sigma VP FEG SEM equipped with a Gatan 3View2 system, where datasets of up to 1000 images were acquired of the block surface every 100 nm through automated sectioning. Each image was acquired at 4K × 4K pixels, at a pixel resolution of between 6.5 and 21 nm and a pixel dwell time of 8 μs, using an SEM accelerating voltage of 3.5 keV in low vacuum variable pressure mode (28–30 Pa). Imaging data were acquired from a 26.5 μm × 26.5 μm region of interest. Selected serial image sequences were extracted from the image data and 3D reconstructions were generated using Amira 6.1 software (FEI). For TEM, ultrathin 90 nm sections were taken from the SBF-SEM pins using a Leica UC6 ultramicrotome. The sections were then collected on TAAB G300HEX grids and examined using a JEOL 1010 TEM at an accelerating voltage of 80Kv.

### Statistics and reproducibility

The Alamar Blue proliferation assay of HCEC on the hydrogels was analyzed using a two-way ANOVA (*n*=5 technical replicates (TR) per group). BMDC activation assays were performed using a one-way ANOVA with a Brown-Forsythe test and Tukey’s multiple comparisons test with a confidence interval of 95% for each marker (GraphPad Prism 8.4.2, GraphPad Software, LLC., San Diego, CA, USA). The unit of analysis was the mouse (*n*=6, per group).

The unit of analysis for the clinical statistics was the eye. The clinical statistics were conducted on uneven population sizes (CLP-PEG *n*=4; CLP-PEG-MPC *n*=4; unoperated *n*=8). For variables with repeated measures over time, a mixed-effects analysis with Geisser-Greenhouse’s correction was performed (α=0.05) with a Tukey multiple comparison test for treatment effects by time point (GraphPad Prism 8.4.2). Post-mortem collagen content analysis was performed using the two-way ANOVA with Tukey’s multiple comparisons test. (α=0.05) (IMB^®^ SPSS^®^ Statistics Version 25, IMB Corp., Armonk, NY, USA). Ordinal data for histopathological assessments of corneal epithelial hyperplasia and neovascularization were analyzed by Mann–Whitney U. Statistical significance was set at p≤0.05.

All graphs were prepared using GraphPad Prism v9.0.2. Data are displayed as mean with individual data points or mean ± standard error of the mean.

### Reporting summary

Further information on research design is available in the Nature Research Reporting Summary linked to this article.

## Supplementary information

Supplementary Information

Reporting Summary

## Data Availability

The quantitative datasets and full GLP mini-pig report generated during the current study are available in the Figshare repository (10.6084/m9.figshare.14251088.v1)^45^. The image datasets generated during the current study are available from the corresponding author on reasonable request.

## References

[1] Mori DN, Kreisel D, Fullerton JN, Gilroy DW, Goldstein DR (2014). Inflammatory triggers of acute rejection of organ allografts. Immunol. Rev..

[2] Oliva MS, Schottman T, Gulati M (2012). Turning the tide of corneal blindness. Indian J. Ophthalmol..

[3] Qazi Y, Hamrah P (2013). Corneal allograft rejection: immunopathogenesis to therapeutics. J. Clin. Cell. Immunol..

[4] Williams KA (2006). How effective is penetrating corneal transplantation? Factors influencing long-term outcome in multivariate analysis. Transplantation.

[5] Gain P (2016). Global survey of corneal transplantation and eye banking. JAMA Ophthalmol..

[6] Yorston D, Garg P (2009). Corneal grafting: what Eye care workers need know. Community Eye Heal. J..

[7] Stacy RC, Jakobiec FA, Michaud NA, Dohlman CH, Colby KA (2011). Characterization of retrokeratoprosthetic membranes in the Boston type 1 keratoprosthesis. Arch. Ophthalmol..

[8] Frick C, Dietz AC, Merritt K, Umbreit TH, Tomazic-Jezic VJ (2006). Effects of prosthetic materials on the host immune response: evaluation of polymethyl-methacrylate (PMMA), polyethylene (PE), and polystyrene (PS) particles. J. Long.-Term. Eff. Med. Implant..

[9] Robert MC, Dohlman CH (2014). A review of corneal melting after Boston Keratoprosthesis. Semin. Ophthalmol..

[10] Črnej A (2016). Effect of penetrating keratoplasty and keratoprosthesis implantation on the posterior segment of the eye. Investig. Ophthalmol. Vis. Sci..

[11] Zhou, G. & Groth, T. Host responses to biomaterials and anti-inflammatory design—a brief review. *Macromol. Biosci*. **18**, e1800112 (2018). 10.1002/mabi.201800112 29920937

[12] Chen S (2010). Characterization of topographical effects on macrophage behavior in a foreign body response model. Biomaterials.

[13] Brodbeck, W. et al. Influence of biomaterial surface chemistry on the apoptosis of adherent cells. *J. Biomed. Mater. Res*. **55**, 661–668 (2001). 10.1002/1097-4636(20010615)55:4<661::aid-jbm1061>3.0.co;2-f 11288096

[14] Brodbeck WG (2002). Biomaterial surface chemistry dictates adherent monocyte/macrophage cytokine expression in vitro. Cytokine.

[15] Coster DJ, Jessup CF, Williams KA (2009). Mechanisms of corneal allograft rejection and regional immunosuppression. Eye.

[16] Di Zazzo A, Kheirkhah A, Abud TB, Goyal S, Dana R (2017). Management of high-risk corneal transplantation. Surv. Ophthalmol..

[17] Niederkorn JY (2010). High-risk corneal allografts and why they lose their immune privilege. Curr. Opin. Allergy Clin. Immunol..

[18] Fagerholm P (2010). A biosynthetic alternative to human donor tissue for inducing corneal regeneration: 24-month follow-up of a phase 1 clinical study. Sci. Transl. Med..

[19] Fagerholm P (2014). Stable corneal regeneration four years after implantation of a cell-free recombinant human collagen scaffold. Biomaterials.

[20] Mölzer C (2019). Activation of dendritic cells by crosslinked collagen hydrogels (artificial corneas) varies with their composition. J. Tiss. Eng. Regen. Med..

[21] Islam MM (2018). Biomaterials-enabled cornea regeneration in patients at high risk for rejection of donor tissue transplantation. NJP Regen. Med..

[22] Vuorela A, Myllyharju J, Nissi R, Pihlajaniemi T, Kivirikko KI (1997). Assembly of human prolyl 4‐hydroxylase and type III collagen in the yeast Pichia pastoris: formation of a stable enzyme tetramer requires coexpression with collagen and assembly of a stable collagen requires coexpression with prolyl 4‐hydroxylase. EMBO J..

[23] Yang C (2004). Development of a recombinant human collagen-type III based hemostat. J. Biomed. Mater. Res. B: Appl. Biomater..

[24] Sewald, N. & Jakubke, H.-D. *Peptides: Chemistry and Biology* (Wiley-VCH, 2015).

[25] Collier JH, Segura T (2011). Evolving the use of peptides as components of biomaterials. Biomaterials.

[26] O’Leary LER, Fallas JA, Bakota EL, Kang MK, Hartgerink JD (2011). Multi-hierarchical self-assembly of a collagen mimetic peptide from triple helix to nanofibre and hydrogel. Nat. Chem..

[27] Kumar VA (2014). A nanostructured synthetic collagen mimic for hemostasis. Biomacromolecules.

[28] Islam MM (2016). Self-assembled collagen-like-peptide implants as alternatives to human donor corneal transplantation. RSC Adv..

[29] Jangamreddy JR (2018). Short peptide analogs as alternatives to collagen in pro-regenerative corneal implants. Acta Biomater..

[30] Delgado, L. et al. Collagen cross-linking increases scaffold stability while modulates pro-inflammatory macrophage response. *Front. Bioeng. Biotechnol*. **4**, (2016).

[31] Samarawickrama C (2018). Collagen-based fillers as alternatives to cyanoacrylate glue for the sealing of large corneal perfortaions. Cornea.

[32] Cho HK (2011). Preparation and characterization of MRI-active gadolinium nanocomposite particles for neutron capture therapy. J. Mater. Chem..

[33] Pahlitzsch T, Sinha P (1985). The alkali burned cornea: electron microscopical, enzyme histochemical, and biochemical observations. Graefe’s Arch. Clin. Exp. Ophthalmol..

[34] Ishihara K, Ishikawa E, Iwasaki Y, Nakabayashi N (1999). Inhibition of fibroblast cell adhesion on substrate by coating with 2-methacryloyloxyethyl phosphorylcholine polymers. J. Biomater. Sci. Polym. Ed..

[35] McRae Page S (2014). Promoting cell adhesion on slippery phosphorylcholine hydrogel surfaces. J. Mater. Chem. B.

[36] Rehany U, Waisman M (1994). Suppression of corneal allograft rejection by systemic cyclosporine-A in heavily vascularized rabbit corneas following alkali burns. Cornea.

[37] Sun M (2011). Collagen V is a dominant regulator of collagen fibrillogenesis: dysfunctional regulation of structure and function in a corneal-stroma-specific Col5a1-null mouse model. J. Cell Sci..

[38] Hackett JM (2011). Biosynthetic corneal implants for replacement of pathologic corneal tissue: performance in a controlled rabbit alkali burn model. Invest. Ophthalmol. Vis. Sci..

[39] Wong ML, Griffiths LG (2014). Immunogenicity in xenogeneic scaffold generation: antigen removal vs. decellularization. Acta Biomater..

[40] Food and Drug Administration, C. for B. E. and R. Source Animal, Product, Preclinical, and Clinical Issues Concerning the Use of Xenotransplantation Products in Humans; Guidance for Industry. http://www.fda.gov/BiologicsBloodVaccines/GuidanceComplianceRegulatoryInformation/Guidances/default.htm. (2003).

[41] Haagdorens M (2019). In vitro cultivation of limbal epithelial stem cells on surface-modified crosslinked collagen scaffolds. Stem Cells Int..

[42] Araki-Sasaki K (1995). An SV40-immortalized human corneal epithelial cell line and its characterization. Invest. Ophthalmol. Vis. Sci..

[43] Islam MM (2015). Functional fabrication of recombinant human collagen-phosphorylcholine hydrogels for regenerative medicine applications. Acta Biomater..

[44] Islam MM (2019). Effects of gamma radiation sterilization on the structural and biological properties of decellularized corneal xenografts. Acta Biomater..

[45] Simpson, F. C. et al. Collagen analogs with phosphorylcholine serve as inflammation-suppressing scaffolds for corneal regeneration from burns in mini-pigs—Raw Chart Data. *Figshare Dataset* (2021) 10.6084/m9.figshare.14251088.v1 (2021) 10.1038/s42003-021-02108-y PMC8140136 34021240

[46] Théry C (2018). Minimal information for studies of extracellular vesicles 2018 (MISEV2018): a position statement of the International Society for Extracellular Vesicles and update of the MISEV2014 guidelines. J. Extracell. Vesicles.

[47] Deerinck T (2010). Enhancing serial block-face scanning electron microscopy to enable high resolution 3-D nanohistology of cells and tissues. Microsc. Microanal..

[48] Zeng Y, Yang J, Huang K, Lee Z, Lee X (2001). A comparison of biomechanical properties between human and porcine cornea. J. Biomech..

[49] Crabb RAB, Chau EP, Evans MC, Barocas VH, Hubel A (2006). Biomechanical and microstructural characteristics of a collagen film-based corneal stroma equivalent. Tissue Eng..

[50] Jue B, Maurice DM (1986). The mechanical properties of the rabbit and human cornea. J. Biomech..

[51] Doutch J, Quantock AJ, Smith VA, Meek KM (2008). Light transmission in the human cornea as a function of position across the ocular surface: theoretical and experimental aspects. Biophys. J..

[52] Liu W (2009). Collagen-phosphorylcholine interpenetrating network hydrogels as corneal substitutes. Biomaterials.

[53] Patel S, Tutchenko L (2019). The refractive index of the human cornea: a review. Cont. Lens Anterior Eye.

[54] Maurice, D. M. in *The Eye* (ed. Davson, H.) 525 (Elsevier Science, 1984).

