# Supplementary material for: Collagen analogs with phosphorylcholine are inflammation-suppressing scaffolds for corneal regeneration from alkali burns in mini-pigs
Source: Commun Biol. 2021 May 21;4:608. doi: 10.1038/s42003-021-02108-y (PMC8140136; doi:10.1038/s42003-021-02108-y)
Supplement: Supplementary file 1 — Supplementary Information [file 42003_2021_2108_MOESM1_ESM.pdf]

Supplementary Information

Collagen analogs with phosphorylcholine are inflammation-suppressing scaffolds for corneal regeneration from alkali burns in mini-pigs

Fiona C. Simpson[1-4], †, Christopher D. McTiernan[5], †, Mohammad Mirazul Islam[6], Oleksiy Buznyk[7,8], Philip N. Lewis[9], Keith M. Meek[9], Michel Haagdorens[10], Cindy Audiger[1,11], Sylvie Lesage[1,11], François-Xavier Gueriot[1,12], Isabelle Brunette[1,2], Marie-Claude Robert[1,4], David Olsen[13], Laura Koivusalo[14], Aneta Liszka[7], Per Fagerholm[7, *], Miguel Gonzalez-Andrades[6, 15,*], May Griffith[1-4, *]

[1]Centre de recherche, Hôpital Maisonneuve-Rosemont, Montréal, QC, Canada
[2]Départment d'Ophtalmologie, Université de Montréal, Montréal, QC, Canada
[3]Institut du Génie Biomédicale, Université de Montréal, Montréal, QC, Canada
[4]Centre de recherche du Centre hospitalier de l'Université de Montréal, Montréal, QC, Canada
[5]Division of Cardiac Surgery, University of Ottawa Heart Institute, Ottawa, ON, Canada
[6]Disruptive Technology Laboratory, Massachusetts Eye and Ear and Schepens Eye Research Institute, Department of Ophthalmology, Harvard Medical School, Boston, MA, USA
[7]Institute for Clinical and Experimental Medicine, Linköping University, Linköping, Sweden
[8]Filatov Institute of Eye Diseases and Tissue Therapy of the NAMS of Ukraine, Odessa, Ukraine
[9]School of Optometry and Vision Sciences, Cardiff University, Cardiff, UK
[10]Department of Ophthalmology, Visual Optics and Visual Rehabilitation, University of Antwerp, Antwerp, Belgium
[11]Département de microbiologie, infectiologie et immunologie, Université de Montréal, Montréal, QC, Canada
[12]Department of Ophthalmology, Valence Hospital, France
[13]FibroGen Inc., San Francisco, CA, USA
[14]Faculty of Medicine and Health Technology, Tampere University, Tampere, Finland
[15]Maimonides Biomedical Research Institute of Cordoba (IMIBIC), Department of Ophthalmology, Reina Sofia University Hospital and University of Cordoba, Cordoba, Spain

†These authors contributed equally. * These authors jointly supervised this work

*To Whom Correspondence Should be Addressed:
May.Griffith@umontreal.ca
Per.Fagerholm@liu.se
miguel.gonzalez@imibic.org

Supplementary Notes

Supplementary Note 1: Implantation in a feline cornea

This experiment was conducted in accordance with the ARVO Statement for the Use of Animals in Ophthalmic and Vision Research and with the Maisonneuve-Rosemont Hospital Committee for Animal Protection guidelines.

A CLP-PEG-MPC and a CLP-PEG implant were grafted into the cornea of a male domestic cat (*Felis catus*), 2 years of age. Feline and human corneas share several anatomical and functional characteristics, including comparable corneal thicknesses and curvatures, as well as the inability of their corneal endothelial cells to replicate *in vivo* (contrary to rodents or rabbits), which allows reliable quantification of endothelial damage in case of an eventual toxicity of the biomaterial. The large diameter of the feline cornea (15.5–18 mm) also allows for high precision *in vivo* measurements using the same instrumentation and techniques as for human subjects.

Under general anesthesia, a 350 μm deep limbal incision was made using a diamond-knife followed by dissection of an intrastromal tunnel through which the implant was inserted. Each implant was 4-mm in diameter and 200 μm thick. They were inserted poles apart, CLP-PEG-MPC in the superior cornea and CLP-PEG in the inferior cornea, and in both cases, 2 mm away from the limbus. No stiches were required to maintain the intrastromal implants and the two limbal incisions were closed using two 10-0 nylon sutures. Post-operative medication consisted of prednisone 5 mg PO daily for 3 days, as well as topical moxifloxacin 0.5% BID, tobramycin 0.3% - dexamethasone 0.1% BID, and diclofenac BID for one month.

A complete eye examination, including slit-lamp photos, intraocular pressure measurement, ultrasound central pachymetry, and noninvasive, noncontact anterior segment optical coherent tomography (Visante 1000, Carl Zeiss Meditec, Lena, Germany), was performed daily during the first week, twice a week for the first month, and then monthly until 14 months after surgery. Corneal aesthesiometry to quantify touch sensitivity, specular microscopy of the corneal endothelium (Konan Medical Inc., Nishinomiya, Japan), as well as corneal surface and 3D-shape analyses (Pentacam HR, Oculus Inc., Wetzlar, Germany) were also performed before and 3, 6, 9, and 12 months after surgery.

Corneal epithelial surface integrity was maintained at all times and the implants remained clear throughout the entire 14-month postoperative period (Figure S1, S2). On the second post-operative week, a granular haze appeared at the stromal-implant interface, around, in front and behind the implants, reaching a maximum at three months post-operation. Stromal neovascularisation to the CLP-PEG-MPC implant was also noticed during the third month, reaching a maximum at 5 months (Figure S1 e-f). A low-dose steroid treatment (tobramycin 0.3% - dexamethasone 0.1% BID) was introduced for two months (months 4 to 6). Blood vessels and stromal haze progressively regressed

from months 9 to 14 (Figure S1i-l, S2 a-b). Corneal aesthesiometry showing touch sensitivity, and endothelial cell density remained stable like those of the normal control eye throughout the study. OCT confirmed stable implantation into the host cornea (Figure S3). Corneal topography also demonstrated the ability of these implants to stably remodel the 3D corneal shape (Figure S2 c-d), a property that is interesting for potential corneal reconstruction in diseased corneas.

Supplementary Figures

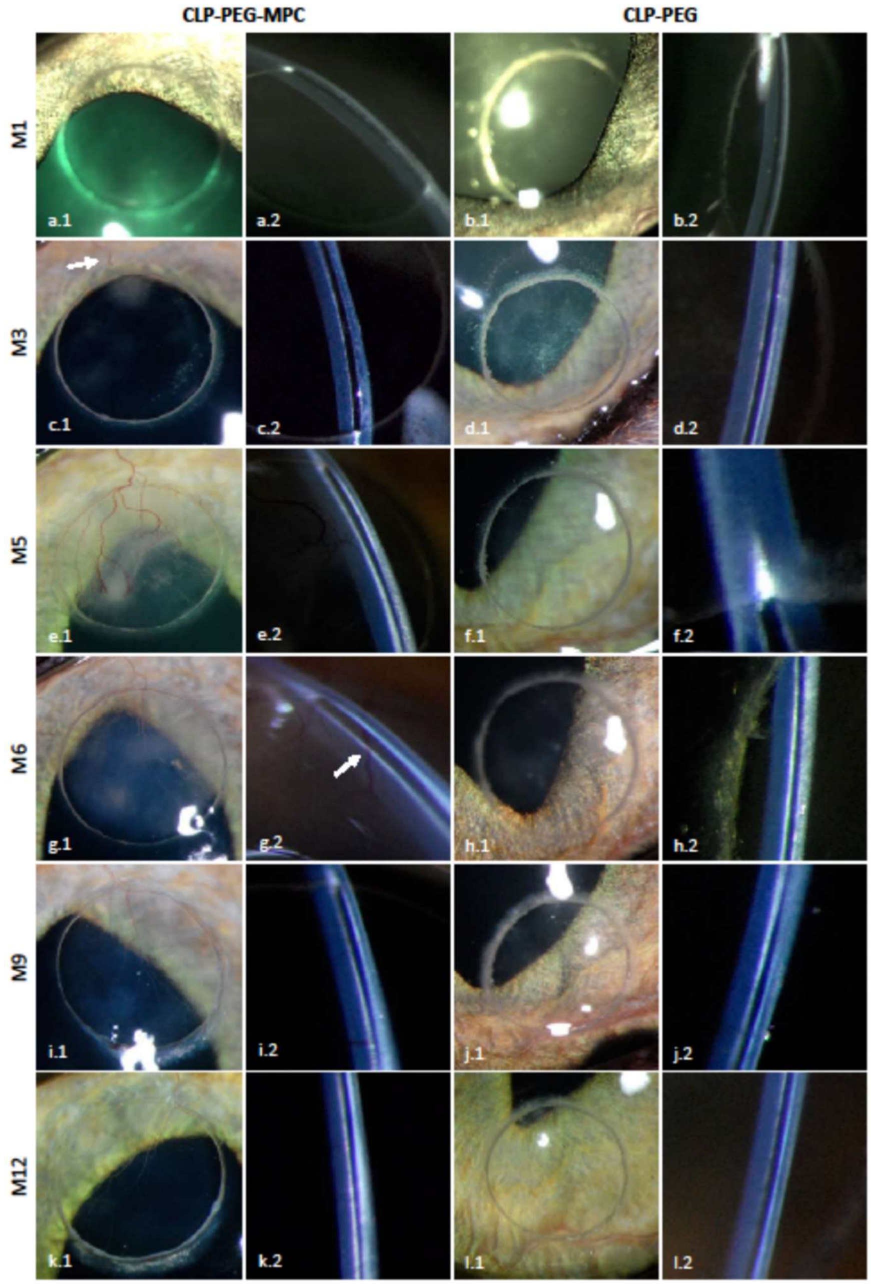

Supplementary Figure 1. Monthly (M1 to M12) slit-lamp follow-up of the feline model implants over a 12-month observation period. At 1-month post-operation, the implants remained transparent as shown in the gross morphology (a1,b1) and corresponding slit lamp images (a2, b2). Maximum haze and neovascularization were seen between M3-6, corresponding to the in-growth of stromal cells (c-g). After six months, both haze and neovascularization regressed so that both implants are transparent at nine months (i, j) and 12 months (k, l) post-operation. The vessel seen in the slit lamp image (g2 – arrowed) was no longer present at 9 or 12 months.

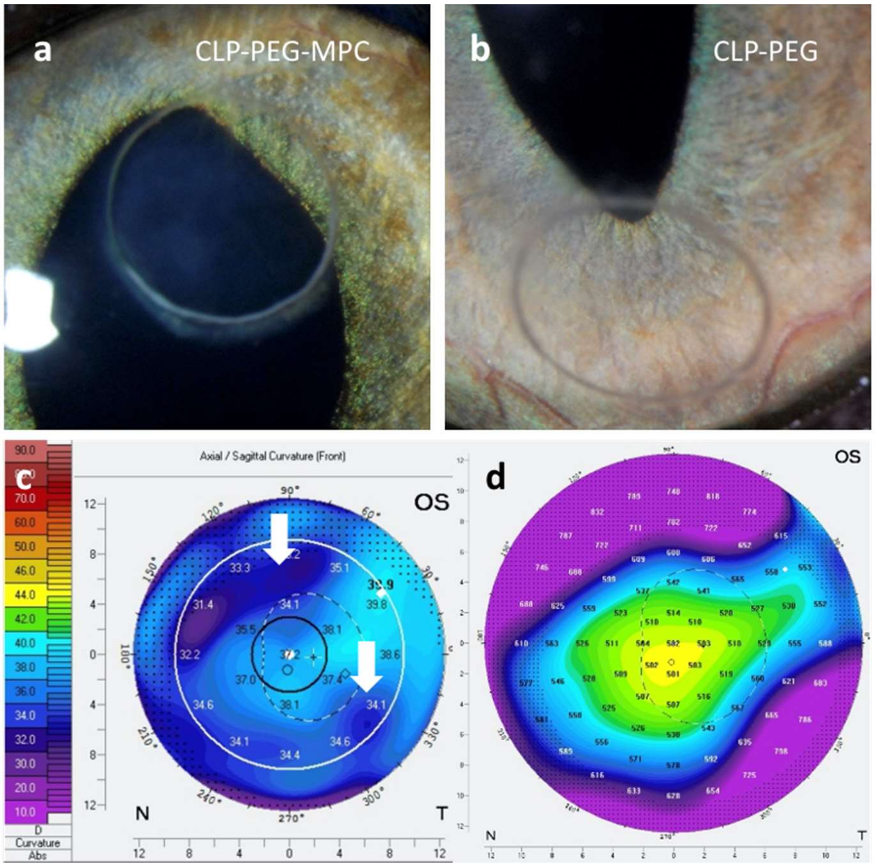

Supplementary Figure 2. Feline corneal transparency at 14 months post-operation, allowing visualization of the fine details of the iris through the implants (a, b). Surface mapping of corneal front surface curvature showed stable flattening induced by the implants (c) while preserving central pachymetry (d).

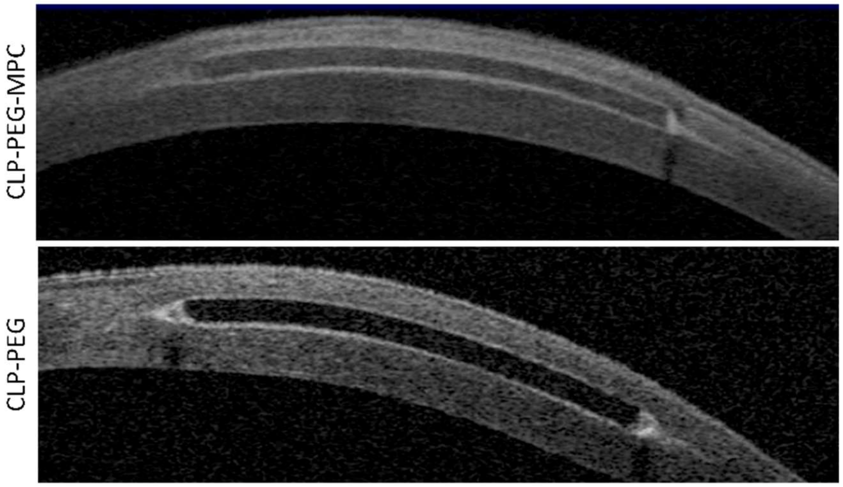

Supplementary Figure 3. Optical coherence tomography (OCT) of the CLP-PEG-MPC (Top) and CLP-PEG (Bottom) implants at one year after implantation in the feline model showing retention of shape and transparency.

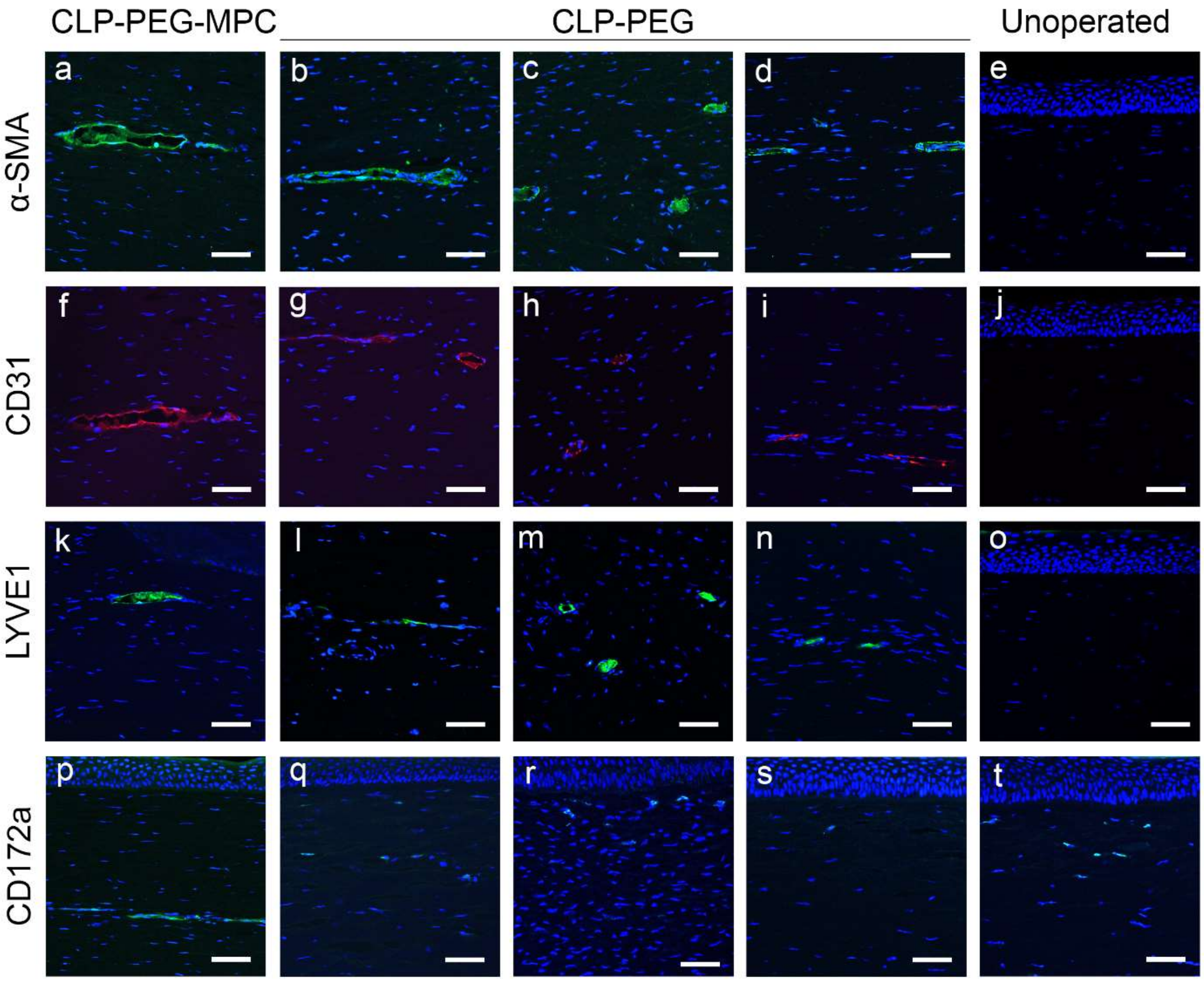

Supplementary Figure 4. Vascular and inflammatory markers in the regenerated mini-pig neo-corneas at 12-month after CLP-PEG-MPC implantation compared to CLP-PEG and controls. One out of four CLP-PEG-MPC pigs showed positive staining (in green) by an antibody against smooth muscle actin (α-SMA) (a), red staining for blood vessel endothelial cell marker, CD31 (f), and green staining for the lymphatic marker, LYVE 1 (k). Three of four CLP-PEG pigs showed positive vascular staining for α-SMA (b-d), CD31 (g-i), and LYVE 1 (l-n). Unoperated corneas expressed no vascular markers. (e,j,o). Both the regenerated corneas (p-s) and unoperated cornea (t) contained CD172a positive (green fluorescence) mononuclear cells.

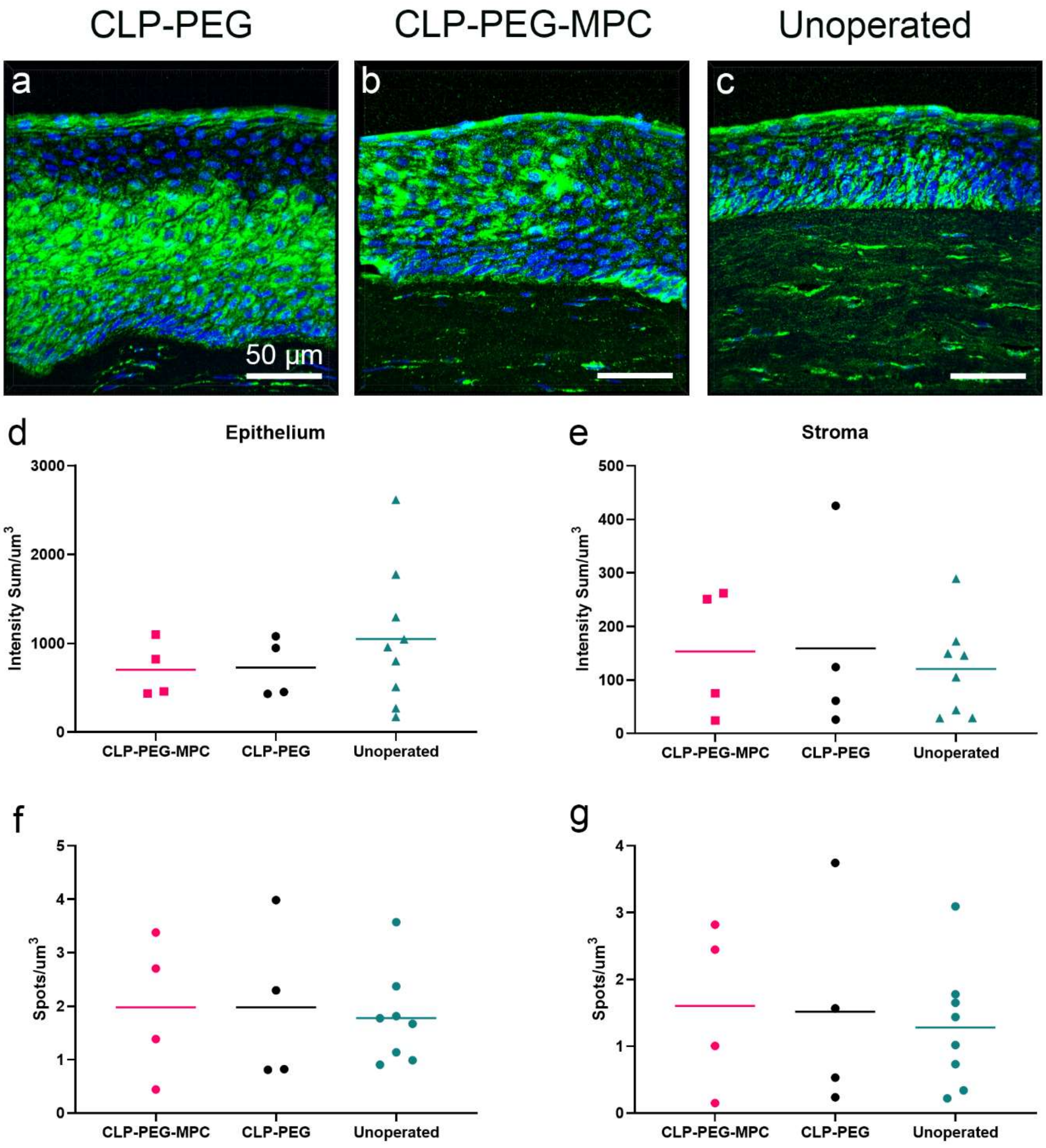

Supplementary Figure 5. Quantification of LL37 expression in CLP-PEG and CLP-PEG-MPC grafted mini-pig corneas at 12 months post-operation. (a-c) 3D reconstructions of confocal images of LL37-stained corneas, counterstained with DAPI. (d-e) Quantification of the intensity sum of spots constructed using LL37 fluorescence by area. (f-g) Quantification of the total number of spots by area.

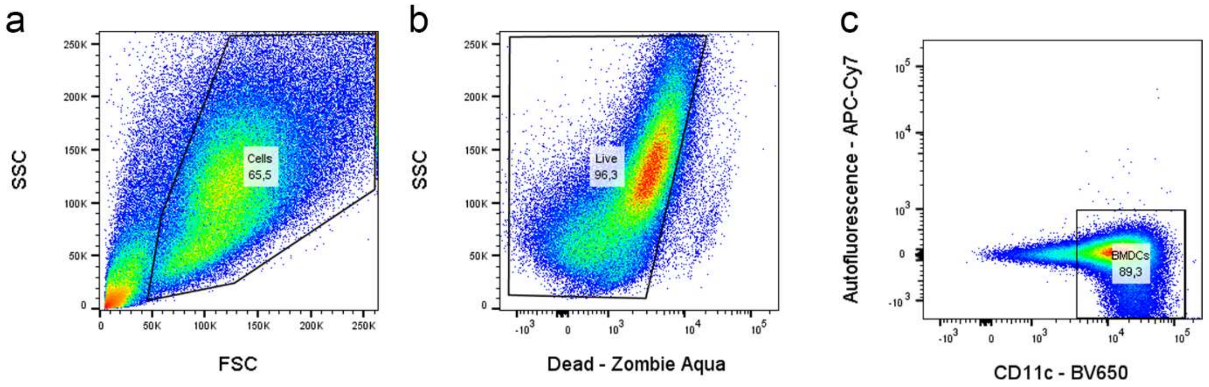

Supplementary Figure 6. Sample flow cytometry gating strategy for bone marrow-derived dendritic cells (BMDCs). The cells were gated for size and granularity using a FSC/SSC gate (a). The cells were gated to remove dead cells, based on low Zombie-Aqua fluorescence in live cells that were impermeable to the dye (b). The live cells were gated for CD11c high, autofluorescence low (c) and this is the gate that was subject to subsequent analysis.

Supplementary Tables

Supplementary Table 1. Two-Way ANOVA of Alamar Blue proliferation study

ANOVA table	SS	DF	MS	F (DFn, DFd)	P value
Time x Treatment	1.37E+13	4	3.42E+12	F (4, 24) = 14.28	P<0.0001
Time	8.22E+14	2	4.11E+14	F (1.163, 13.96) = 1713	P<0.0001
Treatment	2.02E+13	2	1.01E+13	F (2, 12) = 31.96	P<0.0001
Subject	3.8E+12	12	3.17E+11	F (12, 24) = 1.320	P=0.2706
Residual	5.76E+12	24	2.4E+11		

Source of Variation	% of total variation	P value	Geisser-Greenhouse's epsilon
Time x Treatment	1.584	<0.0001	
Time	94.97	<0.0001	0.5815
Treatment	2.339	<0.0001	
Subject	0.4392	0.2706	

Tukey's multiple comparisons test	Mean Diff.	95.00% CI of diff.	Adjusted P Value
**Day 1**			
CLP-PEG vs. CLP-PEG-MPC	355174	-7340 to 717688	0.0544
CLP-PEG vs. Control	-42545	-311772 to 226681	0.8751
CLP-PEG-MPC vs. Control	-397719	-737139 to -58299	0.0281
**Day 4**			
CLP-PEG vs. CLP-PEG-MPC	482553	-108848 to 1073954	0.0991
CLP-PEG vs. Control	-521052	-798324 to -243779	0.0018
CLP-PEG-MPC vs. Control	-1003604	-1596622 to -410587	0.0062
**Day 6**			
CLP-PEG vs. CLP-PEG-MPC	2002578	20439 to 3984717	0.0481
CLP-PEG vs. Control	-1503370	-2490533 to -516208	0.0098
CLP-PEG-MPC vs. Control	-3505949	-5529979 to -1481919	0.0071

Supplementary Table 2. Two-Way ANOVA of Haze

ANOVA table	SS	DF	MS	F (DFn, DFd)	P value
Time x Treatment	4.152	7	0.5932	F (7, 42) = 3.075	P=0.0104
Time	53.65	7	7.665	F (3.082, 18.49) = 39.73	P<0.0001
Treatment	3.754	1	3.754	F (1, 6) = 5.049	P=0.0657
Subject	4.461	6	0.7435	F (6, 42) = 3.854	P=0.0038
Residual	8.102	42	0.1929		

Source of Variation	% of total variation	P value	Geisser-Greenhouse's epsilon
Time x Treatment	0.0657	0.0657	
Time	0.0038	0.0038	0.4403
Treatment	0.0657	0.0657	
Subject	0.0038	0.0038	

Sidak's multiple comparisons test	Mean Diff.	95.00% CI of diff.	Adjusted P Value
**CLP-PEG vs. CLP-PEG-MPC**			
Alkali Burn	0.000		
6 Weeks Post-Burn	0.2500	-1.136 to 1.636	0.9953
Pre-Surgery	-0.2500	-1.829 to 1.329	0.9979
7 Weeks	1.250	-0.3288 to 2.829	0.1315
3 Months	1.250	-1.356 to 3.856	0.5513
6 Months	0.5000	-0.9479 to 1.948	0.8437
9 Months	0.2500	-1.460 to 1.960	0.9811
12 Months	0.6250	-1.013 to 2.263	0.4850

Supplementary Table 3. Two-Way ANOVA of Aesthesiometry

**ANOVA table**	**SS**	**DF**	**MS**	**F (DFn, DFd)**	**P value**
Time x Treatment	18.80	12	1.567	F (12, 78) = 10.72	P<0.0001
Time	31.79	6	5.298	F (1.622, 21.09) = 36.24	P<0.0001
Treatment	11.86	2	5.929	F (2, 13) = 27.67	P<0.0001
Subject	2.786	13	0.2143	F (13, 78) = 1.466	P=0.1496
Residual	11.40	78	0.1462		

**Source of Variation**	**% of total variation**	**P value**	**Geisser-Greenhouse's epsilon**
Time x Treatment	28.61	<0.0001	
Time	48.37	<0.0001	0.2703
Treatment	18.04	<0.0001	
Subject	4.239	0.1496	

**Tukey's multiple comparisons test**	**Mean Diff.**	**95.00% CI of diff.**	**Adjusted P Value**
**Alkali Burn**			
CLP-PEG vs. CLP-PEG-MPC	0.1250	-0.3973 to 0.6473	0.6259
CLP-PEG vs. Unoperated	0.06250	-0.1216 to 0.2466	0.6000
CLP-PEG-MPC vs. Unoperated	-0.06250	-0.5331 to 0.4081	0.8980
**7 Weeks**			
CLP-PEG vs. CLP-PEG-MPC	-0.3750	-3.324 to 2.574	0.9179
CLP-PEG vs. Unoperated	-2.375	-5.495 to 0.7445	0.0983
CLP-PEG-MPC vs. Unoperated	-2.000	-4.413 to 0.4126	0.0802
**3 Months**			
CLP-PEG vs. CLP-PEG-MPC	-0.6250	-1.597 to 0.3472	0.1980
CLP-PEG vs. Unoperated	-1.688	-2.463 to -0.9118	0.0029
CLP-PEG-MPC vs. Unoperated	-1.063	-1.986 to -0.1389	0.0322
**6 Months**			
CLP-PEG vs. CLP-PEG-MPC	0.1250	-0.4643 to 0.7143	0.7969
CLP-PEG vs. Unoperated	-0.7500	-1.353 to -0.1468	0.0280
CLP-PEG-MPC vs. Unoperated	-0.8750	-1.397 to -0.3527	0.0122
**9 Months**			
CLP-PEG vs. CLP-PEG-MPC	0.000	-0.5424 to 0.5424	>0.9999
CLP-PEG vs. Unoperated	0.000	-0.4661 to 0.4661	>0.9999
CLP-PEG-MPC vs. Unoperated	0.000	-0.4661 to 0.4661	>0.9999
**12 Months**			
CLP-PEG vs. CLP-PEG-MPC	-0.2500	-0.8532 to 0.3532	0.3292
CLP-PEG vs. Unoperated	-0.2500	-0.8532 to 0.3532	0.3292
CLP-PEG-MPC vs. Unoperated	0.000		

Supplementary Table 4. Two-Way ANOVA of Pachymetry

ANOVA table	SS	DF	MS	F (DFn, DFd)	P value
Time x Treatment	0.7125	4	0.1781	F (4, 24) = 0.4597	P=0.7645
Time	27.59	4	6.897	F (1.280, 7.682) = 17.80	P=0.0023
Treatment	0.5062	1	0.5062	F (1, 6) = 0.8351	P=0.3961
Subject	3.638	6	0.6063	F (6, 24) = 1.565	P=0.2006
Residual	9.300	24	0.3875		

Source of Variation	% of total variation	P value	Geisser-Greenhouse's epsilon
Time x Treatment	9.078	<0.0001	
Time	15.95	<0.0001	0.4514
Treatment	26.69	0.0351	
Subject	39.57	<0.0001	

Tukey's multiple comparisons test	Mean Diff.	95.00% CI of diff.	Adjusted P Value
**Pre-Surgery**			
CLP-PEG vs. CLP-PEG-MPC	31.00	-173.7 to 235.7	0.8332
CLP-PEG vs. Unoperated	90.50	34.61 to 146.4	0.0033
CLP-PEG-MPC vs. Unoperated	59.50	-140.5 to 259.5	0.5607
**3 Months**			
CLP-PEG vs. CLP-PEG-MPC	217.3	-102.1 to 536.6	0.1504
CLP-PEG vs. Unoperated	172.9	27.21 to 318.5	0.0281
CLP-PEG-MPC vs. Unoperated	-44.38	-381.7 to 292.9	0.8668
**6 Months**			
CLP-PEG vs. CLP-PEG-MPC	159.0	-98.86 to 416.9	0.1854
CLP-PEG vs. Unoperated	137.8	32.88 to 242.6	0.0194
CLP-PEG-MPC vs. Unoperated	-21.25	-293.0 to 250.5	0.9486
**9 Months**			
CLP-PEG vs. CLP-PEG-MPC	160.0	-113.8 to 433.8	0.2060
CLP-PEG vs. Unoperated	123.5	20.17 to 226.8	0.0247
CLP-PEG-MPC vs. Unoperated	-36.50	-318.5 to 245.5	0.8762
**12 Months**			
CLP-PEG vs. CLP-PEG-MPC	135.3	-96.61 to 367.1	0.2120
CLP-PEG vs. Unoperated	106.1	8.618 to 203.6	0.0365
CLP-PEG-MPC vs. Unoperated	-29.13	-269.3 to 211.1	0.8905

Supplementary Table 5. Two-Way ANOVA of Schirmer's Tear Test

ANOVA table	SS	DF	MS	F (DFn, DFd)	P value
Time x Treatment	152.9	12	12.75	F (12, 78) = 0.4649	P=0.9292
Time	456.2	6	76.04	F (4.508, 58.60) = 2.774	P=0.0300
Treatment	53.05	2	26.53	F (2, 13) = 0.5568	P=0.5861
Subject	619.4	13	47.64	F (13, 78) = 1.738	P=0.0693
Residual	2139	78	27.42		

Source of Variation	% of total variation	P value	Geisser-Greenhouse's epsilon
Time x Treatment	4.330	0.9292	
Time	12.92	0.0300	0.7513
Treatment	1.502	0.5861	
Subject	17.54	0.0693	

Tukey's multiple comparisons test	Mean Diff.	95.00% CI of diff.	Adjusted P Value
**Alkali Burn**			
CLP-PEG vs. CLP-PEG-MPC	-5.250	-15.29 to 4.792	0.2503
CLP-PEG vs. Unoperated	-1.125	-4.735 to 2.485	0.6720
CLP-PEG-MPC vs. Unoperated	4.125	-5.777 to 14.03	0.3818
**Pre-Surgery**			
CLP-PEG vs. CLP-PEG-MPC	-3.250	-15.46 to 8.956	0.7058
CLP-PEG vs. Unoperated	-0.7500	-11.94 to 10.44	0.9788
CLP-PEG-MPC vs. Unoperated	2.500	-7.821 to 12.82	0.7717
**7 Weeks**			
CLP-PEG vs. CLP-PEG-MPC	-2.000	-14.24 to 10.24	0.8406
CLP-PEG vs. Unoperated	-2.750	-8.582 to 3.082	0.4208
CLP-PEG-MPC vs. Unoperated	-0.7500	-12.79 to 11.29	0.9757
**3 Months**			
CLP-PEG vs. CLP-PEG-MPC	-2.750	-14.31 to 8.813	0.7508
CLP-PEG vs. Unoperated	-1.375	-12.19 to 9.444	0.9185
CLP-PEG-MPC vs. Unoperated	1.375	-7.469 to 10.22	0.8930
**6 Months**			
CLP-PEG vs. CLP-PEG-MPC	-4.000	-14.66 to 6.657	0.5151
CLP-PEG vs. Unoperated	-3.250	-12.08 to 5.583	0.5759
CLP-PEG-MPC vs. Unoperated	0.7500	-9.436 to 10.94	0.9750
**9 Months**			
CLP-PEG vs. CLP-PEG-MPC	-0.2500	-14.58 to 14.08	0.9984
CLP-PEG vs. Unoperated	-1.500	-13.20 to 10.20	0.9239
CLP-PEG-MPC vs. Unoperated	-1.250	-14.23 to 11.73	0.9535
**12 Months**			
CLP-PEG vs. CLP-PEG-MPC	4.250	-10.95 to 19.45	0.6421
CLP-PEG vs. Unoperated	1.875	-13.22 to 16.97	0.9069
CLP-PEG-MPC vs. Unoperated	-2.375	-10.50 to 5.750	0.6879

Supplementary Table 6. Two-Way ANOVA of Intraocular Pressure

ANOVA table	SS	DF	MS	F (DFn, DFd)	P value
Time x Treatment	43.63	10	4.363	F (10, 65) = 0.4729	P=0.9016
Time	55.88	5	11.18	F (2.416, 31.40) = 1.211	P=0.3169
Treatment	100.3	2	50.13	F (2, 13) = 3.589	P=0.0574
Subject	181.6	13	13.97	F (13, 65) = 1.514	P=0.1363
Residual	43.63	10	4.363	F (10, 65) = 0.4729	P=0.9016

Source of Variation	% of total variation	P value	Geisser-Greenhouse's epsilon
Time x Treatment	4.422	0.9016	
Time	5.665	0.3169	0.4831
Treatment	10.16	0.0574	
Subject	18.41	0.1363	

Tukey's multiple comparisons test	Mean Diff.	95.00% CI of diff.	Adjusted P Value
**Alkali Burn**			
CLP-PEG vs. CLP-PEG-MPC	3.250	-10.16 to 16.66	0.6630
CLP-PEG vs. Unoperated	2.500	-10.32 to 15.32	0.7934
CLP-PEG-MPC vs. Unoperated	-0.7500	-6.002 to 4.502	0.9196
**Pre-Surgery**			
CLP-PEG vs. CLP-PEG-MPC	3.000	-0.3856 to 6.386	0.0702
CLP-PEG vs. Unoperated	4.000	0.7067 to 7.293	0.0210
CLP-PEG-MPC vs. Unoperated	1.000	-1.237 to 3.237	0.4501
**3 Months**			
CLP-PEG vs. CLP-PEG-MPC	1.750	-5.370 to 8.870	0.7398
CLP-PEG vs. Unoperated	2.250	-4.611 to 9.111	0.5073
CLP-PEG-MPC vs. Unoperated	0.5000	-5.295 to 6.295	0.9485
**6 Months**			
CLP-PEG vs. CLP-PEG-MPC	2.500	-3.694 to 8.694	0.4585
CLP-PEG vs. Unoperated	0.7500	-5.276 to 6.776	0.9096
CLP-PEG-MPC vs. Unoperated	-1.750	-5.904 to 2.404	0.4540
**9 Months**			
CLP-PEG vs. CLP-PEG-MPC	0.2500	-5.828 to 6.328	0.9912
CLP-PEG vs. Unoperated	0.3750	-4.814 to 5.564	0.9770
CLP-PEG-MPC vs. Unoperated	0.1250	-5.520 to 5.770	0.9977
**12 Months**			
CLP-PEG vs. CLP-PEG-MPC	4.750	0.07843 to 9.422	0.0473
CLP-PEG vs. Unoperated	3.375	-1.096 to 7.846	0.1434
CLP-PEG-MPC vs. Unoperated	-1.375	-6.679 to 3.929	0.7595

Supplementary Table 7. Mann-Whitney U tests of Histopathology Data

	**P value**	**Mean rank of CLP-PEG**	**Mean rank of CLP-PEG-MPC**	**Mean rank diff.**	**Mann-Whitney U**	**q value**
**Epithelial Hyperplasia**	0.485714	5.125	3.875	1.250	5.500	0.981143
**Vascularization**	>0.999999	5.000	4.000	1.000	6.000	>0.999999

Supplementary Table 8. Multiple unpaired t-tests of IHC Quantification

**Multiple Unpaired t-tests of IHC Quantification**				
	α-SMA	CD31	LYVE1	CD172a
P value	0.131512	0.243655	0.18914	0.267544
Mean of CLP-PEG	5.5	5.25	3	10.63
Mean of CLP-PEG-MPC	0.75	1.5	0.5	2
Difference	4.75	3.75	2.5	8.625
SE of difference	2.445	2.704	1.555	6.548
t ratio	1.943	1.387	1.608	1.317
df	3.616	3.677	3.683	3.491
q value	0.270219	0.270219	0.270219	0.270219

Supplementary Table 9. Collagen Content Two-Way ANOVA with Tukey's multiple comparisons test

ANOVA table	SS (Type III)	DF	MS	F (DFn, DFd)	P value
Interaction	2.54976E+13	10	2.54976E+12	F (10, 78) = 10.07	P<0.0001
Collagen Type	1.202E+15	5	2.405E+14	F (5, 78) = 950.0	P<0.0001
Treatment	1.78611E+13	2	8.93053E+12	F (2, 78) = 35.28	P<0.0001
Residual	1.97457E+13	78	2.5315E+11		

Source of Variation	% of total variation	P value
Interaction	1.753	<0.0001
Collagen Type	82.69	<0.0001
Treatment	1.228	<0.0001

Tukey's multiple comparisons test	Predicted (LS) mean diff.	95.00% CI of diff.	Adjusted P Value
**HMW**			
CLP-PEG vs. CLP-PEG-MPC	1116880	266844 to 1966916	0.0067
CLP-PEG vs. Unoperated	966473	230320 to 1702625	0.0067
CLP-PEG-MPC vs. Unoperated	-150407	-886560 to 585746	0.8771
γ			
CLP-PEG vs. CLP-PEG-MPC	664016	-186020 to 1514052	0.1553
CLP-PEG vs. Unoperated	-284861	-1021014 to 451291	0.6265
CLP-PEG-MPC vs. Unoperated	-948877	-1685030 to -212725	0.008
**β**			
CLP-PEG vs. CLP-PEG-MPC	1718101	868065 to 2568136	<0.0001
CLP-PEG vs. Unoperated	-1166399	-1902551 to -430246	0.0009
CLP-PEG-MPC vs. Unoperated	-2884499	-3620652 to -2148347	<0.0001
α1(V)			
CLP-PEG vs. CLP-PEG-MPC	436061	-413975 to 1286097	0.4417
CLP-PEG vs. Unoperated	1092919	356767 to 1829072	0.0019
CLP-PEG-MPC vs. Unoperated	656859	-79294 to 1393011	0.09
**α1(I)**			
CLP-PEG vs. CLP-PEG-MPC	2167571	1317535 to 3017607	<0.0001
CLP-PEG vs. Unoperated	1169005	432853 to 1905158	0.0008
CLP-PEG-MPC vs. Unoperated	-998566	-1734719 to -262413	0.0049
**α2(I)**			
CLP-PEG vs. CLP-PEG-MPC	1077958	227922 to 1927994	0.0092
CLP-PEG vs. Unoperated	807574	71422 to 1543727	0.0281
CLP-PEG-MPC vs. Unoperated	-270384	-1006536 to 465769	0.656

Supplementary Table 10. Antibodies used for flow cytometry

Target	Antibody	Dilution Factor
CD11c	Brilliant Violet 650™ anti-mouse CD11c,(Clone: N418),(IsoType: Armenian Hamster IgG),(Reactivity: Mouse),(Format: BV650),(APP: FC),(Species: Hamster), Biolegend, 117339	1:1600
IA-IE (MHC Class II)	PerCP/Cy5.5 anti-mouse I-A/I-E,(Clone: M5/114.15.2),(IsoType: Rat IgG2b, κ),(Reactivity: Mouse),(Format: PerCP/Cy5.5),(APP: FC),(Species: Rat), Biolegend, 107626	1:3200
CD40	CD40, APC, clone: 1C10, eBioscience™, 501129392	1:400
CD80	PE anti-mouse CD80,(Clone: 16-10A1),(IsoType: Armenian Hamster IgG),(Reactivity: Mouse, Cross-Reactivity: Dog (Canine)),(Format: PE),(APP: FC),(Species: Hamster), Biolegend, 104708	1:100
CD86	FITC anti-mouse CD86,(Clone: GL-1),(IsoType: Rat IgG2a, κ),(Reactivity: Mouse),(Format: FITC),(APP: FC),(Species: Rat), Biolegend, 105006	1:50

Supplementary Table 11. Antibodies used for immunohistochemistry

Target	Antibody (or Lectin)	Dilution Factor
Mucin	Lectin from Ulex europaeus (gorse, furze) FITC conjugate, Sigma-Aldrich, L9006	1:500
Cytokeratin K3/K76	Anti-Keratin K3/K76 Antibody, clone AE5, Millipore, CBL218	1:50
α-SMA	Anti-alpha smooth muscle Actin antibody [1A4], AbCam, ab7817	1:500
LYVE1	Anti-LYVE1 antibody, AbCam, ab33682	1:100
CD172a	Mouse Anti Pig CD172a, Nordic BioSite, ST-MCA2312GA	1:100
Collagen V	Anti-Collagen V antibody, AbCam, ab134800	1:500
CD31	Anti-CD31 antibody, AbCam, ab28364	1:50
LL37	Anti-LL37/Cathelicidin antibody, LSBio, LS-B6696	1:100
Mouse IgG	Alexa Fluor® 488 AffiniPure Goat Anti-Mouse IgG (H+L), Jacskon Immuno Labs, 115-545-146	1:1000
Rabbit IgG	Alexa Fluor® 488 AffiniPure Goat Anti-Rabbit IgG (H+L), Jackson ImmunoResearch Laboratories, 111-545-144	1:1000
CD9	CD9 Mouse anti-Bovine, Canine, Equine, Feline, Human, Mink, Mustelid, Non-human primate, Porcine, Rabbit, Clone: MM2/57, Invitrogen™, MA180307	1:100
Tsg101	Recombinant Anti-TSG101 antibody [EPR7130(B)], AbCam, ab125011	1:100
Rabbit IgG	IgG (H+L) Highly Cross-Adsorbed Goat anti-Rabbit, Alexa Fluor® 594, Invitrogen, A11037	1:1000
Mouse IgG	IgG (H+L) Highly Cross-Adsorbed Goat anti-Mouse, Alexa Fluor® Plus 647, Invitrogen™, PIA32728	1:1000
