# Supplementary material for: Collagen analogs with phosphorylcholine are inflammation-suppressing scaffolds for corneal regeneration from alkali burns in mini-pigs
Source: Commun Biol. 2021 May 21;4:608. doi: 10.1038/s42003-021-02108-y (PMC8140136; doi:10.1038/s42003-021-02108-y)
Supplement: Supplementary file 2 — Reporting Summary [file 42003_2021_2108_MOESM2_ESM.pdf]

nature research

Corresponding author(s): May Griffith, Per Fagerholm, Miguel Gonzalez-Andrades

Last updated by author(s): Mar 3, 2021

Reporting Summary

Nature Research wishes to improve the reproducibility of the work that we publish. This form provides structure for consistency and transparency in reporting. For further information on Nature Research policies, see our Editorial Policies and the Editorial Policy Checklist.

Statistics

For all statistical analyses, confirm that the following items are present in the figure legend, table legend, main text, or Methods section.

n/a	Confirmed	
[ ]	[x]	The exact sample size (*n*) for each experimental group/condition, given as a discrete number and unit of measurement
[ ]	[x]	A statement on whether measurements were taken from distinct samples or whether the same sample was measured repeatedly
[ ]	[x]	The statistical test(s) used AND whether they are one- or two-sided<br>*Only common tests should be described solely by name; describe more complex techniques in the Methods section.*
[x]	[ ]	A description of all covariates tested
[ ]	[x]	A description of any assumptions or corrections, such as tests of normality and adjustment for multiple comparisons
[ ]	[x]	A full description of the statistical parameters including central tendency (e.g. means) or other basic estimates (e.g. regression coefficient) AND variation (e.g. standard deviation) or associated estimates of uncertainty (e.g. confidence intervals)
[ ]	[x]	For null hypothesis testing, the test statistic (e.g. *F*, *t*, *r*) with confidence intervals, effect sizes, degrees of freedom and *P* value noted<br>*Give P values as exact values whenever suitable.*
[x]	[ ]	For Bayesian analysis, information on the choice of priors and Markov chain Monte Carlo settings
[x]	[ ]	For hierarchical and complex designs, identification of the appropriate level for tests and full reporting of outcomes
[x]	[ ]	Estimates of effect sizes (e.g. Cohen's *d*, Pearson's *r*), indicating how they were calculated

Our web collection on statistics for biologists contains articles on many of the points above.

Software and code

Policy information about availability of computer code

Data collection

Instron Series IX/S, Instron, Norwood, MA
Trios, TA Instruments, New Castle, DE, USA
Spectramax M2e, Molecular Devices, San Jose, CA, USA
Wallac Viktor2 1420 Multilabel counter , PerkinElmer, Waltham, MA, USA
FACSDiva, Becton, Dickinson & Company, Franklin Lakes, NJ, USA
Heidelberg Eye Explorer (HEYEX), Heidelberg, Germany
GE Healthcare Image Quant 350, GE Healthcare, Chicago, IL, USA
Zen Blue, Carl Zeiss Microscopy, Göttingen, Germany
JEOL 1010 TEM, JEOL USA, Inc., Peabody, MA
Gatan 3View2, Carl Zeiss Microscopy, Göttingen, Germany

Data analysis	Microsoft Office Excel,<br>GraphPad Prism 9.0.2, GraphPad Software, LLC., San Diego, CA, USA<br>IBM® SPSS® Statistics Version 25, IMB Corp., Armonk, NY, USA<br>Imaris v9.2.1 (Bitplane Inc., Concord, MA, USA)<br>FIJI, Schindelin, J.; Arganda-Carreras, I. & Frise, E. et al. (2012), "Fiji: an open-source platform for biological-image analysis", Nature methods 9 (7): 676-682<br>FlowJo, Becton, Dickinson & Company, Franklin Lakes, NJ, USA<br>Mnova, Mestrelab Research, Santiago de Compostela, SPAIN

For manuscripts utilizing custom algorithms or software that are central to the research but not yet described in published literature, software must be made available to editors and reviewers. We strongly encourage code deposition in a community repository (e.g. GitHub). See the Nature Research guidelines for submitting code & software for further information.

Data

Policy information about availability of data

All manuscripts must include a data availability statement. This statement should provide the following information, where applicable:

- Accession codes, unique identifiers, or web links for publicly available datasets
- A list of figures that have associated raw data
- A description of any restrictions on data availability

All data needed to evaluate the conclusions in the paper are present in the paper and/or the Supplementary Materials.

Field-specific reporting

Please select the one below that is the best fit for your research. If you are not sure, read the appropriate sections before making your selection.

[x] Life sciences [ ] Behavioural & social sciences [ ] Ecological, evolutionary & environmental sciences

For a reference copy of the document with all sections, see nature.com/documents/nr-reporting-summary-flat.pdf

Life sciences study design

All studies must disclose on these points even when the disclosure is negative.

Sample size	No samples size calculations were performed for this study. The number of pigs per group (n=4) is a standard amount for safety and toxicology testing.
Data exclusions	No data was excluded from this study.
Replication	This study has not been replicated.
Randomization	The pigs were randomly allocated to the two biomaterials groups by the veterinary team at Adlego.
Blinding	The corneal surgeons were blinded as to which of the CLP-PEG or CLP-PEG-MPC implants were implanted in each pig cornea.

Reporting for specific materials, systems and methods

We require information from authors about some types of materials, experimental systems and methods used in many studies. Here, indicate whether each material, system or method listed is relevant to your study. If you are not sure if a list item applies to your research, read the appropriate section before selecting a response.

Materials & experimental systems

n/a	Involved in the study	
[ ]	[x]	Antibodies
[ ]	[x]	Eukaryotic cell lines
[x]	[ ]	Palaeontology and archaeology
[ ]	[x]	Animals and other organisms
[x]	[ ]	Human research participants
[x]	[ ]	Clinical data
[x]	[ ]	Dual use research of concern

Methods

n/a	Involved in the study	
[x]	[ ]	ChIP-seq
[ ]	[x]	Flow cytometry
[x]	[ ]	MRI-based neuroimaging

Antibodies

Antibodies used	All antibodies used in the manuscript are listed in Tables S10 and S11.
Validation	Antibodies for IHC were titrated in normal pig cornea cryosections based on the manufacturers recommended dilution factors in comparison to secondary-only and no antibody controls. Antibodies for flow-cytometry were titrated in immature and mature bone-marrow derived dendritic cells and the expression of each marker was compared between the two phenotypes. All antibodies were titrated based on the manufacturer's recommended dilution factors.

Eukaryotic cell lines

Policy information about cell lines

Cell line source(s)	The immortalized SV40 HCECs were a gift from H Handa, Division of Ophthalmology, Kinki Central Hospital, Hyogo, Japan. A stable GFP-HCEC cell line was established by the transfection of the SV40 immortalized HCEC cells with a vector containing a puromycin-resistant gene together with GFP, using the Lipofectamine 2000 Transfection Reagent (Life Technologies, California, USA). Selection of puromycin-resistant cells with 2 ug ml–1 of puromycin added to the medium was performed to obtain stable GFP-expressing lines.
Authentication	The initial immortalized HCEC line was characterized using the expression of keratin and large T antigen (Araki-Sasaki et al. Invest Ophthalmol. Vis. Sci. 1995;36(3):614-621.) GFP-HCECs were subsequently characterized by morphology, and expression of Integrin Beta1 and focal adhesion kinase cell proliferation rate (Islam et al. Acta Biomater. 2015;12:70-80.).
Mycoplasma contamination	These cells were not tested for mycoplasma contamination.
Commonly misidentified lines (See ICLAC register)	N/A

Animals and other organisms

Policy information about studies involving animals; ARRIVE guidelines recommended for reporting animal research

Laboratory animals	Mini-pigs: Sus scrofa domesticus, Göttingen, female, approx. 8 months old at start of study<br>Mice: Mus musculus, C57BL/6J, male, 6-12 weeks<br>Cat: Felis catus (domestic cat) , adult male, 2 years
Wild animals	N/A
Field-collected samples	N/A
Ethics oversight	The mini pig study was performed in compliance with the Swedish Animal Welfare Ordinance and the Animal Welfare Act, with ethical permission from the local ethical committee in Stockholm (N209/15), and in accordance with OECD Principles of Good Laboratory Practices (GLP), ENV/MC/CHEM (98) 17, 1997, by Adlego Biomedical AB (Stockholm, Sweden). The cat and mice experiments were conducted in accordance with the ARVO Statement for the Use of Animals in Ophthalmic and Vision Research and with the Maisonneuve-Rosemont Hospital Committee for Animal Protection guidelines.

Note that full information on the approval of the study protocol must also be provided in the manuscript.

Flow Cytometry

Plots

Confirm that:

- [x] The axis labels state the marker and fluorochrome used (e.g. CD4-FITC).
- [x] The axis scales are clearly visible. Include numbers along axes only for bottom left plot of group (a 'group' is an analysis of identical markers).
- [x] All plots are contour plots with outliers or pseudocolor plots.
- [x] A numerical value for number of cells or percentage (with statistics) is provided.

Methodology

Sample preparation	Bone marrow was isolated from the femur and tibia of 6 to 12-week-old, male C57BL/6J mice. Cells (1x10^6/well) were seeded onto 6-well suspension culture plates in RPMI 1640 containing 10% (v/v) fetal bovine serum (Wisent, Saint-Jean-Baptiste, QC), 0.5 mg/mL penicillin-streptomycin-glutamine, 10 mM HEPES, 1 mM sodium pyruvate, 55 µm β-mercaptoethanol and 25 ng/mL granulocyte-macrophage colony-stimulating factor (GM-CSF) (Gibco, Waltham, MA). Complete RPMI, containing 50 ng/mL GM-CSF, was exchanged for half of the media on days two and three of culture. Cultures were maintained for six days, then collected and subject to density gradient centrifugation using Histodenz™ (Sigma-Aldrich, St. Louis, MO) to separate the enlarged BMDCs. The selected cells were seeded at a density of 1x10^6 cells/well on a 24 well plate for materials testing.<br>Hydrogel components (EDC/NHS, DMTMM, CLP, CLP-PEG, and MPC) were applied to the BMDCs at an equivalent total mass

	to a 10 mm, 500 μm thick hydrogel disk, to simulate the total amount present in a complete corneal implant. (Hydrogels disks (6mm, 500 μm thick) were incubated with BMDCs for 24 hours. Lipopolysaccharide was used as a positive control.
Instrument	All samples were collected using a BD LSR II flow cytometer
Software	Collection was conducted using BD FACS Diva software. Analysis was conducted using FlowJo software and Graph Pad Prism.
Cell population abundance	Mature dendritic cells composed 50-99% of the live gate. (The low 50% is due to cell death from exposure to toxic crosslinkers).
Gating strategy	The cells were gated for size and granularity using a FSC/SSC gate (Fig S6a). The cells were gated to remove dead cells, based on low Zombie-Aqua (Fig S6b). The live cells were gated for CD11c high, autofluorescnece low (Fig S6c) and this is the gate that was subject to subsequent analysis.

☒ Tick this box to confirm that a figure exemplifying the gating strategy is provided in the Supplementary Information.
